# Defects in lysosomal function and lipid metabolism in human microglia harboring a *TREM2* loss of function mutation

**DOI:** 10.1007/s00401-023-02568-y

**Published:** 2023-04-28

**Authors:** Fabia Filipello, Shih-Feng You, Farzaneh S. Mirfakhar, Sidhartha Mahali, Bryan Bollman, Mariana Acquarone, Olena Korvatska, Jacob A. Marsh, Anirudh Sivaraman, Rita Martinez, Claudia Cantoni, Luca De Feo, Laura Ghezzi, Miguel A. Minaya, Arun Renganathan, Anil G. Cashikar, Jun-Ichi Satoh, Wandy Beatty, Abhirami K. Iyer, Marina Cella, Wendy H. Raskind, Laura Piccio, Celeste M. Karch

**Affiliations:** 1 Department of Psychiatry, Washington University in St Louis, St Louis, MO USA; 2 Department of Psychiatry and Behavioral Sciences, University of Washington, Seattle, WA USA; 3 Department of Medicine, Division of Medical Genetics, University of Washington, Seattle, WA USA; 4 Department of Neurology, Washington University in St Louis, St Louis, MO USA; 5 Department of Bioinformatics and Molecular Neuropathology, Meiji Pharmaceutical University, Tokyo, Japan; 6 Department of Molecular Microbiology, Washington University School of Medicine, St. Louis, MO 63110 USA; 7 Department Of Pathology and Immunology, Washington University in St Louis, St Louis, MO USA; 8 Charles Perkins Centre and Brain and Mind Centre, School of Medical Sciences (Neuroscience), University of Sydney, Sydney, NSW Australia; 9 School of Medical Sciences, Brain and Mind Centre, University of Sydney, 94 Mallett St, Camperdown, Sydney, NSW 2050 Australia

**Keywords:** TREM2, Induced pluripotent stem cells, Microglia, Nasu-Hakola disease, Transcriptomics, Lysosome

## Abstract

TREM2 is an innate immune receptor expressed by microglia in the adult brain. Genetic variation in the *TREM2* gene has been implicated in risk for Alzheimer’s disease and frontotemporal dementia, while homozygous *TREM2* mutations cause a rare leukodystrophy, Nasu-Hakola disease (NHD). Despite extensive investigation, the role of TREM2 in NHD pathogenesis remains poorly understood. Here, we investigate the mechanisms by which a homozygous stop-gain *TREM2* mutation (p.Q33X) contributes to NHD. Induced pluripotent stem cell (iPSC)-derived microglia (iMGLs) were generated from two NHD families: three homozygous *TREM2* p.Q33X mutation carriers (termed NHD), two heterozygous mutation carriers, one related non-carrier, and two unrelated non-carriers. Transcriptomic and biochemical analyses revealed that iMGLs from NHD patients exhibited lysosomal dysfunction, downregulation of cholesterol genes, and reduced lipid droplets compared to controls. Also, NHD iMGLs displayed defective activation and HLA antigen presentation. This defective activation and lipid droplet content were restored by enhancing lysosomal biogenesis through mTOR-dependent and independent pathways. Alteration in lysosomal gene expression, such as decreased expression of genes implicated in lysosomal acidification (*ATP6AP2*) and chaperone mediated autophagy (*LAMP2*), together with reduction in lipid droplets were also observed in post-mortem brain tissues from NHD patients, thus closely recapitulating in vivo the phenotype observed in iMGLs in vitro. Our study provides the first cellular and molecular evidence that the *TREM2* p.Q33X mutation in microglia leads to defects in lysosomal function and that compounds targeting lysosomal biogenesis restore a number of NHD microglial defects. A better understanding of how microglial lipid metabolism and lysosomal machinery are altered in NHD and how these defects impact microglia activation may provide new insights into mechanisms underlying NHD and other neurodegenerative diseases.

**Supplementary Information:**

The online version contains supplementary material available at 10.1007/s00401-023-02568-y.

## Introduction

Nasu-Hakola disease (NHD), also referred to as polycystic lipomembranous osteodysplasia with sclerosing leukoencephalopathy (PLOSL; OMIM 221770, OMIM 605086), is a rare autosomal recessive disorder characterized by a progressive presenile dementia and bone cysts. Approximately 200 NHD cases have been reported worldwide, primarily in Japan and Finland, with few cases also reported in Italy [2, 7, 39, 44, 69, 82, 96].

Clinically, patients with NHD show recurrent pathological bone fractures, with frontal lobe syndrome and progressive dementia during the fourth decade of life [63, 77]. Pathologically, the brains of NHD patients exhibit extensive demyelination, astrogliosis, axonal loss, accumulation of axonal spheroids, calcification, and activation of microglia in the white matter of frontal and temporal lobes and the basal ganglia [3, 77]. Cortical deposition of amyloid beta (Aβ) and focal neocortical neurofibrillary pathology have been also described [33]. NHD is caused by homozygous pathogenic mutations in the genes *triggering receptor expressed on myeloid cells 2* (*TREM2*) or *TYRO protein tyrosine kinase binding protein* (*TYROBP*), alternatively named *DNAX-activation protein 12* (*DAP12*) [78, 79, 83]. Seventeen homozygous pathogenic mutations have been identified in *TREM2* or *DAP12* genes and are predicted to cause loss of function and lead to a similar disease phenotype [50, 52, 57, 105]. More recently, missense heterozygous variants in *TREM2* have been implicated in risk for late-onset Alzheimer’s disease (AD) [37, 42] and frontotemporal dementia [9, 38]. Thus, understanding the functional impact of *TREM2* gene variants has major implications for a number of dementias.

The TREM2 receptor is expressed in myeloid cell populations, including peripheral macrophages and microglia in the central nervous system (CNS) [47, 91]. TREM2 is a type I trans-membrane glycoprotein with an extracellular V-type immunoglobulin (Ig) ectodomain, a connecting stalk followed by a transmembrane region and a C-terminal tail [51]. At the cell surface, TREM2 receptor signaling occurs through association with the adaptor protein DAP12, which interacts with the transmembrane domain of TREM2. The TREM2 receptor has been shown to bind bacterial components [19], anionic and zwitterionic lipids [102], and myelin [88]; yet, the endogenous ligand remains poorly defined. Activation of the TREM2/DAP12 complex mediates the recruitment of the protein tyrosine kinase Syk, resulting in the phosphorylation of downstream mediators such as PLC-γ, PI3K and Vav2/3, which lead to mTOR and mitogen-activated protein kinase (MAPK)-mediated downstream signaling [16, 85, 101]. These intracellular signals promote macrophage/microglia survival [75, 76, 102]; proliferation [76]; phagocytosis [97]; synapse elimination [28]; cellular metabolism [101]; and autophagy [101]. Proteolytic cleavage of the TREM2 ectodomain and/or translation of an alternatively spliced *TREM2* transcript [20, 27, 41, 68, 95, 103] are responsible for the production of a soluble form of the receptor (sTREM2) which is detected in the cerebrospinal fluid (CSF) [27, 86]. Some of the NHD-causing missense mutations in *TREM2* likely affect TREM2 protein folding and stability [53], but the cellular mechanisms and the pathways dysregulated during the disease are still unknown. Despite the predicted loss of function effect of NHD mutations in *TREM2*, mouse models of *Trem2* deficiency fail to capture key pathological aspects of NHD. As such, there remains a critical gap in our understanding of the pathologic impact of NHD mutations in the CNS and our ability to develop novel therapeutics.

Human stem cell models have emerged as a powerful cellular system that enables modeling of rare mutations in the cell-types affected in disease, including those that cause NHD [12, 18, 32, 73]. Yet, the extent to which stem cell models capture disease relevant phenotypes remains uncertain. To begin to define the impact of a rare loss of function mutation in *TREM2* on microglia function, we generated macrophages and human induced pluripotent stem cell (iPSC)-derived microglialike cells (iMGLs) from two families affected by NHD. The three affected individuals are homozygous for the *TREM2* p*.*Q33X mutation, two unaffected heterozygous *TREM2* p.Q33X parents, a healthy sibling that is homozygous for the wild-type allele (*TREM2* WT), and two unrelated controls (*TREM2* WT). *TREM2* p.Q33X introduces an early stop codon and is subject to nonsense mediated decay; hence, there is no TREM2 protein product [80]. Herein, transcriptional profiling and functional analyses of the *TREM2* p.Q33X mutation reveal that it leads to dysregulation of lysosomal function, lipid metabolism and microglia activation, and these phenotypes are recapitulated in brain tissues from NHD patients. Finally, we find that targeting lysosomal dysfunction in an mTOR-dependent or independent manner rescues lysosomal, lipid and activation state defects caused by the *TREM2* mutation.

## Materials and methods

### Patient consent

Skin biopsies were collected following written informed consent from the control unaffected sibling or the parents for the NHD affected individuals. The informed consent was approved by the Washington University School of Medicine Institutional Review Board and Ethics Committee (IRB 201104178).

### Human postmortem brain tissue

Human tissue sections were obtained from two NHD patients, three control patients and two MS patients. The two MS patients were obtained from The Neuroinflammatory Disease Tissue Repository at Washington University St. Louis. Brain autopsies from NHD tissues were obtained from the University of Washington Neuropathology Core Brain Bank and the Meiji Pharmaceutical University, Tokyo, Japan. Under protocols approved by the Institutional Review Boards of the University of Washington and the Meiji Pharmaceutical University, all patients had previously given informed consent to share and study autopsy material. All methods for processing and analyzing the brain autopsy tissues followed relevant guidelines and regulations. Demographic and clinical characteristics of the donors of human brain tissues at the time of collection are indicated in Supplementary Table 1, online resource.

### Dermal fibroblast isolation

Dermal fibroblasts were isolated from skin biopsies obtained from research participants from two NHD families. Briefly, skin biopsies were collected by surgical punch and stored in Fibroblast Growth Media (Lonza). To isolate dermal fibroblasts from skin biopsy, the biopsies were rinsed with PBS and cut lengthwise with dissecting scissors. The resulting tissue sections were then plated into a dry 24-well tissue culture treated plate (approximately 6–12 sections). After removing excess PBS from the wells, 300µL of fibroblast growth media (Lonza) was carefully added and tissue was incubated at 37°C and 5% CO_2_. After 24 h, tissue was supplemented with 1 mL fibroblast growth media and media changes were repeated every 3–4 days. Fibroblast cells were observed to migrate from the tissue within 2 weeks of culture. Dermal fibroblasts were maintained in Fibroblast Growth Media (Lonza) supplemented with penicillin/streptomycin.

### Macrophage differentiation from PBMCs

Peripheral blood mononuclear cells (PBMCs) (IDE: SB CTRL, IDO: NHD1, and 1F1: NHD2) were purified from human blood on Ficoll-Paque PLUS density gradient (Amersham Biosciences, Piscataway, NJ). To generate macrophages, PBMCs were cultured in 6-well culture plates (3 × 10^6^ cell/well) in RPMI-1640 without fetal bovine serum. After 2 h of culture, PBMCs were washed twice with PBS 1X and cultured in RPMI supplemented with 50 ng/mL MCSF for 7 days at 37°C with 5% CO_2_.

### iPSC generation and characterization

Human fibroblasts (IDE: SB CTRL, F12455: NR CTRL, F14532: NR CTRL2, IDO: NHD1, and 1F1: NHD2, F21675: NHD3, F21673: HET1, F21674: HET2) were transduced with non-integrating Sendai virus carrying the four factors required for reprogramming into iPSC: OCT3/4, SOX2, KLF4, and cMYC [6, 98]. Single colonies showing morphological evidence of reprogramming were isolated by manual dissection. Human iPSCs were cultured using feeder-free conditions (Matrigel, BD Biosciences, Franklin Lakes, NJ, USA). Human iPSCs were thawed (1–2 × 10^6^ cells/mL), diluted in DMEM/F12, and centrifuged at 750 rpm for 3 min. The resulting iPSC pellet was then diluted in mTeSR1 supplemented with Rock inhibitor (Y-27632; 10 µM final). iPSCs were subsequently cultured in 37°C, 6% CO_2_ with daily medium changes (mTesR1, STEMCELL Technologies, Vancouver, BC, CA). The cell lines were regularly tested for mycoplasma. All iPSC lines were characterized using standard methods [98]. Each line was analyzed for chromosomal abnormalities by karyotyping (Supplementary Fig. 1a, b, online resource), for pluripotency markers (OCT4A, SOX2, SSEA4, TRA1) by immunocytochemistry (ICC) (Invitrogen A24881) (Supplementary Fig. 1c, d, online resource), and for *TREM2* mutation status (homozygous and heterozygous for the *TREM2* p.Q33X mutation) by Sanger sequencing (Supplementary Fig. 1e, f, online resource).

### iPSC-derived microglia-like cells (iMGLs)

iMGLs were generated as previously described in [1, 25, 66]. iPSCs were differentiated into hematopoietic precursors cells (HPCs) using a STEMdiff Hematopoietic kit (STEMCELL Technologies) and following manufacturer’s instructions. Briefly, to begin HPC differentiation, iPSCs were detached with ReLeSR™ (STEMCELL Technologies) and passaged in mTeSR1 supplemented with Rock inhibitor to achieve a density of 100–200 aggregates/well (for iPSCs from mutation carrying donors, aggregate numbers to be plated requires line-specific optimization). On day 0, cells were transferred to Medium A from the STEMdiff Hematopoietic Kit. On day 3, flattened endothelial cell colonies were exposed to Medium B and cells remained in Medium B for 10 additional days while HPCs began to lift off the colonies. After 12 days in culture, HPCs were collected by removing the floating population with a serological pipette and the adherent portion after incubation with Accutase™ (STEMCELL Technologies) for 15 min at 37°C. The floating and adherent CD43^+^ population (see below *Fluorescent Activated Cell Sorting (FACS)* section) was sorted with a Becton Dickinson FACSAria II cell sorter. At this point, HPCs can be frozen in CryoStor® CS10 (STEMCELL Technologies) and stored in liquid nitrogen. iMGL induction was achieved by culturing CD43^+^ HPCs in iPSC-Microglia medium (DMEM/F12, 2X insulin-transferrin-selenite, 2X B27, 0.5X N2, 1X glutamax, 1X non-essential amino acids, 400 mM monothioglycerol, and 5 mg/mL human insulin) freshly supplemented with 100 ng/mL IL-34, 50 ng/mL TGFβ1, and 25 ng/mL M-CSF (Peprotech) for 25 days (37 days from iPSC). During the last 3 days in culture, 100 ng/mL CD200 (Novoprotein) and 100 ng/mL CX3CL1 (Peprotech) were added to iPSC-Microglia medium to mimic a brain-like environment (Supplementary Fig. 2A, online resource). All the subsequent analyses described in the paper have been performed on fully mature iMGL (between day 40 to day 52) unless otherwise stated. All the analyses performed in this work were run on the first NHD family (IDE: SB CTRL, F12455: NR CTRL, IDO: NHD1, and 1F1: NHD2) in parallel, and on the second NHD family (F14532: NR CTRL2, F21675: NHD3, F21673: HET1, F21674: HET2) in parallel, except for RNAseq, electron microscopy and LPS treatment, which were run on IDE: SB CTRL, IDO: NHD1, and 1F1: NHD2.

### Fluorescent activated cell sorting (FACS)

All steps were performed on ice or using a pre-chilled refrigerated centrifuge set to 4˚C with all buffers/solutions pre-chilled before addition to samples. For HPC sorting, HPCs were collected using sterile filtered FACS buffer (1X DPBS, 2% BSA), cells were then filtered through 70 μm filters to remove large clumps, washed with FACS buffer (300 × g for 5 min 18 C), then stained for 20 min at 4˚C in the dark using the following antibodies: CD43-APC (Cat#: 343206, Clone: 10G7), CD34-FITC (Cat#: 343504, Clone: 581), CD45-Alexa 700 (Cat#: 304024; Clone: H130) from BioLegend, and Zombie Aqua™ Fixable Viability Kit (BioLegend). The CD43^+^ total population was sorted with a Becton Dickinson FACSAria II cell sorter. For detection of microglial surface markers, iMGLs were incubated for 10 min on ice with anti-CD16/CD32 to block Fc receptors (1:50; Miltenyi Biotec, Cat #:120-000-442) and with Zombie Aqua™ to identify live cells. Then, iMGLs were stained with CD11b-PeCy7 (Cat#: 101216; Clone: M1/70), CD45-Alexa 700 (Cat#: 304024; Clone: H130), CD80-BV421 (Cat#: 305222, Clone: 2D10), CD86-PerCP/Cy5.5 (Cat#: 30420, Clone: IT2.2), MERTK (Cat#: 367620, clone: 590H11G1E3) all from BioLegend, CD14-PeCy7 (Invitrogen, Cat#: 25-0149-41, Clone: 61D3), HLA-DR-PECF594 (BD, Cat#: 562331 clone: G46-6), TREM2-APC (R&D, FAB17291A), TREM2-biotinilated (Clone: E29E3, generously provided by Dr. Marco Colonna) for 20 min at 4˚C in the dark. AnnixinV/Propidium iodide positive cells were detected with a FITC Annexin V Apoptosis Detection Kit with PI (Biolegend). Cells were acquired on a BD LSRFortessa and BDX20 and data analyzed with FlowJo software (FlowJo).

### Lysosomal acidity measurement

To evaluate acidic vesicles, iMGLs were incubated with 5 nM of LysoTracker® Red DND-99 (ThermoFisher, L7528), diluted in the cell medium at 37°C for 5 min. Live cell images of NR CTRL, SB CTRL, NHD1 and NHD2 were acquired with a Nikon Eclipse 80i fluorescent microscope and Metamorph Molecular Devices software. Live cell images of NR CTRL2, HET1, HET2 and NHD3 were acquired with Zeiss LSM980 Airyscan 2 laser scanning confocal microscope (Carl Zeiss Inc., Thornwood, NY) at 40x. A microtubule probe (ViaFluor® 488, Biotium, 70062-T) was used to define cellular structure. Fluorescence intensity of LysoTracker Red was quantified by Fiji. Briefly, the fluorescence intensity of LysoTracker Red in the soma of each cell was measured and then corrected for background fluorescence resulting in the Corrected Total Cell Fluorescence (CTCF) values. To study the overall protease activity, iMGLs were incubated for 4 h at 37°C with 10 µg/mL of DQ™ Red BSA (ThermoFisher, D12051) diluted in the cell medium. Cells were washed once with HBSS solution. Live and fixed cell images were acquired with the laser confocal microscope ZEISS LSM 980 with Airyscan. Fluorescence intensity of DQ™ Red BSA staining in the soma was quantified by Fiji as previously described (Marwaha and Sharma, 2017). DQ™ Red BSA stained cells were next fixed for further immunofluorescence analyses.

### Myelin production and iMGL treatment

Human myelin was prepared as previously described [15, 71] and stored in lyophilized form at – 80°C. Prior to use, myelin was suspended in DMEM to a final concentration of 2 mg/mL and dissolved by vortexing and sonicating. Myelin was then irradiated with 10,000 RADS to achieve sterility. Aliquots were stored at – 80°C for further use. For treatment with myelin, iMGLs were plated on Matrigel-coated 12-mm coverslips at 5 × 10^4^ cell/well and incubated with 10 μg/mL of pHrodo-labeled myelin (pHrodo™ Red, SE, ThermoFisher) for 24 h.

### Torin1 and curcumin analog C1 treatment in iMGLs

Fully mature iMGLs were plated in 24-well plate at a density of 1 × 10^5^ cell/well and treated with 250 nM Torin 1 (Tocris, 4247) for 4 h or with 1 μM of curcumin analog C1 (Cayman Chemical Company, 34255) for 7 h or with DMSO as untreated control. After 4 h and 7 h of treatment respectively, iMGLs were washed once in PBS and cultured in complete iPSC-Microglia medium for 24 h. Cells were then collected and stained for detection of microglial surface markers by FACS or for PLIN2 by immunocytochemistry (ICC).

### Immunocytochemistry

Cells were washed three times with DPBS (1X) and fixed with cold PFA (4% w/v) for 20 min at room temperature (RT) followed by three washes with PBS (1X). Cells were blocked with blocking solution (PBS with 0.1% Triton X-100 and 1% BSA) for 30 min at RT. Primary antibodies were added at respective dilutions (see below) in blocking solution and placed at 4°C overnight. The next day, cells were washed 3 times with PBS for 5 min and stained with Alexa Fluor conjugated secondary antibodies from Invitrogen (1:800) for 2.5 h at room temperature in the dark. After secondary staining, cells were washed 3 times with PBS and cover slipped with ProLong™ Diamond Antifade Mountant or Fluoromount-G™ (ThermoFisher). Primary antibodies used for ICC: anti-P2ry12 (1:500, HPA014518 Sigma), anti-TREM2 (1:200, AF1828 R&D Systems), anti-CD68 (1:100, M0718 Dako), anti-TMEM119 (1:100, ab185333 Abcam), anti-IBA1 (1:500; Wako, 019-19741).

### Lipid droplets staining

For Perilipin 2 (PLIN2) staining, cells were blocked in PBS with 0.1% saponin and 1% BSA for 5 min and subsequently incubated with ADRP/Perilipin 2 antibody (1:500, 15294-1-AP Proteintech) in PBS with 0.1% saponin and 1% BSA overnight at 4°C. For BODIPY staining, cells were incubated in PBS with BODIPY FL Dye (1:1000 from a 1 mg/mL stock solution in DMSO; ThermoFisher) a dye that specifically labels neutral lipids [89] and Hoechst 33342 (1:5000; ThermoFisher) for 20 min at room temperature (RT). To analyze the percentage of lipid-droplet-containing iMGLs, the numbers of total Hoechst + cells and of Hoechst + cells with BODIPY + lipid droplets were counted, and the percentage of BODIPY + iMGLs was calculated. For confocal analysis, images were acquired with a Zeiss LSM980 Airyscan 2 laser scanning confocal microscope (Carl Zeiss Inc., Thornwood, NY) equipped with 63X and 40X, 1.4 numerical aperture (NA) Zeiss Plan Apochromat oil objectives. The system was equipped with a unique scan head, incorporating a high-resolution galvo scanner along with two PMTs and a 32-element spectral detector. ZEN 3.4.9 Blue edition software was used to obtain Z-stacks through the entire height of the cells. Images taken were optimized for 1 airy unit using the 405 nm diode, 488 nm diode, and 561 nm diode and 633 nm HeNe (helium neon) lasers. Images were finally processed with ImageJ and Imaris Software (Bitplane, Switzerland). Quantification was performed on original orthogonal Z-projections generated in ImageJ software. The particle numbers were quantified with ImageJ 1.5j8 (NIH) with size (pixel2) settings from 0.1 to 10 and circularity from 0 to 1. PLIN2-positive droplets with intensity above cytoplasmic background and size (pixel2) from 10 were quantified. A total of 40–50 cells per line were analyzed.

### Histology and immunohistochemistry of formalin-fixed human brain tissue

Formalin-fixed paraffin embedded (FFPE) 5 μm human tissue sections were stained with solochrome cyanine to detect area of demyelination. For immunohistochemistry analysis, sections were deparaffinized and rehydrated in xylene, then sequential concentrations of ethanol to water followed by antigen retrieval in boiling 0.01 M citric acid pH 6.0 for 10 min. Sections were then incubated in PBS with 5% horse serum and 0.1% triton-X for 1 h at RT followed by incubation overnight at 4°C with primary antibodies: anti-Iba1 (1:250; Novus, NB100-1028), anti-TMEM119 (1:100, ab185333 Abcam), anti-PLIN2 (1:100; 15294-1-AP, Proteintech), anti-CD68 (1:50, M0718 Dako), and monoclonal anti-LAMP1 (H4A3, undiluted, developed by Developmental Studies Hybridoma Bank). After primary antibody incubation, sections were washed three times in PBS and incubated in PBS with Alexa Fluor conjugated secondary antibodies from Invitrogen (1:1000) for 1 h at RT in the dark. Sections were mounted with Vectashield (Vector Laboratories, H-1000). Quantitative evaluation of microglial cell morphology in tissue sections was performed as described in the literature using ramification index (RI) calculated by the following equation: 4π × cell area/ (cell perimeter)^2^ [13]. The RI of perfectly round cells is 1; if morphology deviates from circular form, RI is smaller than 1; when the cell is highly ramified the RI is close to zero. The images were acquired with a Nikon Eclipse Ni fluorescent and bright field microscope equipped with 10X, 20X, and 60X zoom objectives. LAMP1 and PLIN2 were analyzed based on the percent area of LAMP1 + Iba1 + and PLIN2 + Iba1 + staining (number of positive pixels/mm2) and then normalized to the percentage of Iba1 + (number of positive pixels/mm2) within the region of interest. CD68 was analyzed as the percentage of CD68 + TMEM119 + Iba1 + and then normalized based on the percentage of TMEM119 + Iba1 + staining (with the assistance of NIS-Elements software).

### Immunoblotting

iMGLs were lysed in RIPA buffer (50mMTris, 150 mM NaCl, 1% SDS, and 1% Triton X-100) containing PMSF, leupeptin, activated sodium orthovanidate, apoprotinin, and phosphatase inhibitor cocktail 3 (Sigma Aldrich Cat. Number P0044). Lysates were mixed with 4 × Laemmli sample buffer (Bio-Rad, Cat#: 161-0747) and 10% β-mercaptoethanol and heated at 95 °C for 10 min and run on a 4–12% bis–tris gel (Nupage). Proteins were transferred to PVDF membrane and blocked for 1 h at RT in 5% milk in phosphate buffered saline with 0.1% Tween 20 (PBS-T). Membranes were probed with the mouse anti-TREM-2 mAbs supernatants (clones 10B11 and 21E10, undiluted) and GAPDH (1:500, Thermo Fisher Scientific, Cat# MA5-15738, RRID: AB_10977387) overnight at 4°C. Membranes were subsequently washed and incubated in affiniPure Goat anti-mouse HRP (1:2000, Jackson Immuno Research Labs, Cat# 115-035-174, RRID: AB_2338512) for 1 h at RT, washed, and developed using Lumigen ECL ultra-reagent (TMA-100).

### sTREM2 ELISA

A sandwich ELISA for human soluble TREM2 (sTREM2) was developed as previously described [21]. Briefly, an anti-human TREM2 monoclonal antibody (R&D Systems, catalog no. MAB1828, clone 263602; 0.5 mg/ml) was used as a capture antibody and coated overnight at 4°C on MaxiSorp 96-well plates (Nalgene Nunc International, Rochester, NY) in sodium bicarbonate coating buffer (0.015 M Na2CO3 and 0.035 M NaHCO3, pH 9.6). Washes between the steps were done four times with PBS/0.05% Tween 20 (Sigma-Aldrich). Wells were then blocked for 4 h at 37°C with 10% fetal bovine serum (FBS) in phosphate-buffered saline (PBS). Freshly thawed supernatants and recombinant human TREM2 standard (SinoBiological, catalog no. 11084-H08H-50) were incubated in duplicate overnight at 4°C. For detection, a goat anti-human TREM2 biotinylated polyclonal antibody (R&D Systems, catalog # BAF1828; 0.2 mg/mL) was diluted in assay buffer (PBS/10% FBS at 1:3000) and incubated for 1.25 h at RT on an orbital shaker. After washing, wells were incubated with horseradish peroxidase–labeled streptavidin (BD Biosciences, San Jose, CA; diluted 1:3000) for 1 h at RT with orbital shaking. Horseradish peroxidase visualization was performed with 3,3′,5,5′ tetramethylbenzidine (Sigma-Aldrich, St. Louis, MO) added to each well for 10 min at RT in the dark. Color development was stopped by adding an equal volume of 2.5 N H2SO4. Optical density of each well was determined at 450 nm. Samples were run in duplicate in each assay. Raw values are provided as [pg/ml].

### RNA extraction, sequencing, and transcript quantification

Total RNA was isolated from iMGLs cells using the RNeasy Mini Kit (Qiagen, Hilden, Germany) according to the manufacturer’s protocol. For iMGLs cell pellets frozen in Qiazol, 200µL of chloroform was added, then the samples were shaken for 5 s and centrifuged at 12,000 g for 15 min or until complete phase separation. 400-600µL aqueous phase was then transferred into a new tube and cleaned using reagents and spin columns from the RNeasy Mini Kit. Cell line samples were DNase-treated using reagents from the RNase-Free DNase Set (Qiagen, Hilden, Germany). TapeStation 4200 System (Agilent Technologies) was used to perform quality control of the RNA concentration, purity, and degradation based on the estimated RNA integrity Number (RIN), and DV200. Extracted RNA (10 µg) was converted to cDNA by PCR using the High-Capacity cDNA Reverse Transcriptase kit (Life Technologies). Samples were sequenced by an Illumina HiSeq 4000 Systems Technology with a read length of 1 × 150 bp, and an average library size (mapped reads) of 36.5 ± 12.2 million reads per sample.

Identity-by-Descent (IDB) [11] and FastQC analyses were performed to confirm sample identity. STAR (v.2.6.0) [22] was used to align the RNA sequences to the human reference genome: GRCh38.p13 (hg38). The quality of RNA reads including the percentage of mapped reads and length of sequence fragments was performed. Aligned sequences were processed using SAMtools (v.1.9) [59]. The average percentage of unique mapped reads in the BAM files was 82.5% ± 3.62, and the average percentage of mapped reads to GRCh38 was 89.77% ± 5.12. Salmon (v. 0.11.3) [81] was used to quantify the expression of the genes annotated. Protein coding genes were selected for further analyses (Supplementary Table 2, online resource).

### Principal component analyses and differential expression analyses

Human macrophages and iMGLs were plotted using the plotly package [60] based on regularized-logarithm transformation (rlog) counts and utilizing the 500 most variable protein coding genes. Differential gene expression between WT and NHD carriers was calculated using the DESeq2 (v.1.22.2) package [62]. The transcripts per million (TPM) of the gene set of interest were made into heatmaps with ComplexHeatmap [36]. In iMGLs, the TPM was further normalized by the expression of *GAPDH*. The top 500 genes were made into a PCA graph to investigate the differences between samples. The DEG analyses compare the expression between WT and NHD carriers in different cell types and were further made into a volcano plot with ggplot2. The significant DEGs (FDR < 0.05, unless otherwise stated) were split into upregulated and downregulated DEG, and separately searched for pathway enrichment by EnrichR [14, 56, 104].

### RNA expression analysis by NanoString nCounter

RNA was isolated from FFPE sections, as previously described [54]. Briefly, for each sample, four 10 micron sections were processed using the Recover All RNA isolation kit (Ambion, ThermoFisher). RNA yield and fragment-length distribution were measured by Qubit Fluorimeter (Molecular Probes, ThermoFisher) and 2100 Bioanalyzer (Agilent Technologies). 1 μg of total RNA was hybridized with the PanCancer Immune panel (NanoString Technologies). Counts were normalized and log2 transformed using the nSolver 3.0 Analysis Software (NanoString Technologies).

### Transmission electron microscopy

For ultrastructural analyses, iMGLs were fixed in 2% paraformaldehyde/2.5% glutaraldehyde (Ted Pella Inc., Redding, CA) in 100 mM sodium cacodylate buffer, pH 7.2 for 2 h at RT. Samples were washed in sodium cacodylate buffer and postfixed in 2% osmium tetroxide (Ted Pella Inc) for 1 h at RT. After rinsing extensively in dH_2_O, samples were then bloc stained with 1% aqueous uranyl acetate (Electron Microscopy Sciences, Hatfield, PA). Samples were washed in dH_2_O, dehydrated in a graded series of ethanol, and embedded in Eponate 12 resin (Ted Pella Inc). Sections of 95 nm were cut with a Leica Ultracut UCT ultramicrotome (Leica Microsystems Inc., Bannockburn, IL), stained with uranyl acetate and lead citrate, and viewed on a JEOL 1200 EX transmission electron microscope (JEOL USA Inc., Peabody, MA) equipped with an AMT 8 megapixel digital camera and AMT Image Capture Engine V602 software (Advanced Microscopy Techniques, Woburn, MA). Multivesicular bodies (MVB) and lipid droplets analyses were performed with ImageJ (Fiji) and the number of MVB and lipid droplets was counted per cell area.

## Results

### Generation of iMGLs from control and NHD donors

**Fig. 1:**
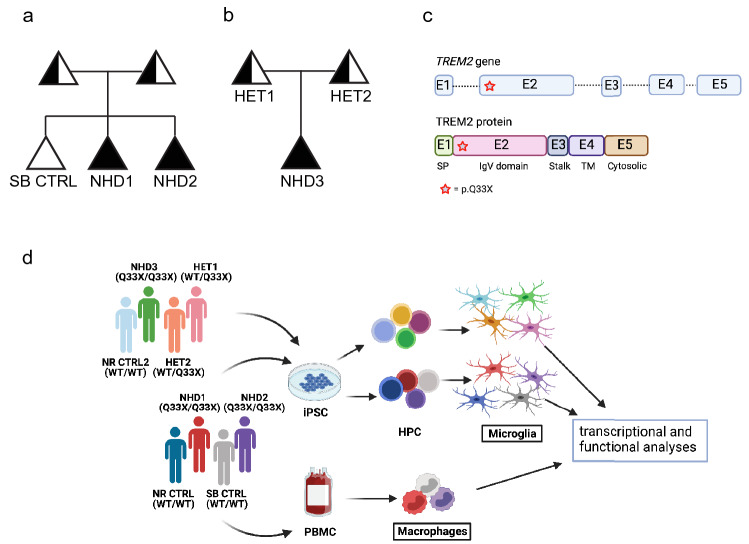
*TREM2* p.Q33X variant in two families affected by NHD. **a** and **b** NHD family pedigrees. Black symbols denote individuals clinically affected by NHD who carry the homozygous *Q33X* mutation in *TREM2* (NHD1, NHD2, and NHD3). White symbols denote a clinically healthy, non-carrier family member (SB CTRL). Half shaded symbols denote heterozygous *TREM2 p.Q33X* carriers (HET1 and HET2). Analyses presented in this manuscript were performed on the three siblings belonging to the first NHD family (SB CTRL, NHD1 and NHD2) and one unrelated control (NR CTRL). For the NHD second family, analyses were performed on two parents (HET1 and HET2), their affected child (NHD3), and one unrelated control (NR CTRL2). Triangles are used to anonymize pedigree. **c** Schematic representation of the human *TREM2* gene (top) and protein (bottom). *TREM2* gene showing exonic (numbered boxes) and intronic (line) regions. The *Q33X* mutation is located in exon 2 (indicated as a red star). **d** Study design depicting the bioinformatic and cellular analyses performed in this study. iPSC-derived human microglia (iMGLs) were generated from iPSCs (all donor lines), while human macrophages were purified from peripheral blood mononuclear cells (PBMCs)-derived from the same donors (NHD1, NHD2 and SB CTRL). Fully differentiated iMGLs and macrophages were assayed for molecular and biochemical analyses

To understand the contribution of a *TREM*2 mutation to myeloid cell dysfunction in NHD, dermal fibroblasts were obtained from two families with reported NHD cases [7, 33]. The first family consisted of two siblings affected by NHD and homozygous for the *TREM2 p.Q33X* mutation (referred to as NHD1 and NHD2) and a third sibling who is homozygous for *TREM2* WT (referred to as SB CTRL; Fig. 1a, c and Supplementary Table 1, online resource). An unrelated control was also analyzed in parallel [45] (referred to as NR CTRL). The main findings were then replicated in a second family composed of three patients, one carrying the *TREM2 p.Q33X* mutation and affected by NHD (referred to as NHD3) and parents heterozygous for *TREM2* p.Q33X (referred to as HET1 and HET2) with an unremarkable clinical history for neurological disorders. Another unrelated control was analyzed in parallel (referred to as NR CTRL2) (Fig. 1b and c and Supplementary Table 1, online resource). Fibroblasts were reprogrammed into iPSC and characterized for pluripotency, genomic stability and for *TREM2* mutation status (Fig. 1d; Supplementary Fig. 1a–f, online resource). Blood samples from the three siblings in the first NHD family were collected, and PBMCs were isolated to generate macrophages, a type of peripheral myeloid cells (Fig. 1d, bottom).

Microglia are the resident macrophages of the brain and the primary cell-type of myeloid origin in the CNS that expresses TREM2. To define the impact of *TREM2* p.Q33X on microglia gene expression and function, we generated iMGLs using a two-step differentiation protocol as previously described [1, 66] (Fig. 1d and Supplementary Fig. 2a, online resource). This method generates iMGLs that are positive for the microglial markers TMEM119, P2RY12, IBA1, and TREM2 (Supplementary Fig. 2b, online resource). iMGLs release sTREM2 in the media, which increases over time in culture and peaks when iMGLs are fully mature (Supplementary Fig. 2c, online resource). iMGLs are distinct from peripheral macrophages derived from PBMCs and express intermediate CD45 (CD45^int^) levels compared to higher levels (CD45^high^) observed in macrophages (Supplementary Fig. 2d, online resource). Additionally, iMGLs produce a gene expression signature that is distinct from macrophages based on principal component analysis of transcriptomic data (Supplementary Fig. 2e, online resource). iMGLs are also enriched for microglia-specific genes including *CX3CR1*, *TGFBR1*, *P2RY12*, *GPR34* and *IRF8* [31, 107] compared with macrophages (fold change > 20; Supplementary Fig. 2f and Supplementary Table 2, online resource). Thus, the microglia differentiation protocol allows us to generate a functionally distinct myeloid population similar to CNS microglia, consistent with previous reports [1, 66].

**Fig. 2:**
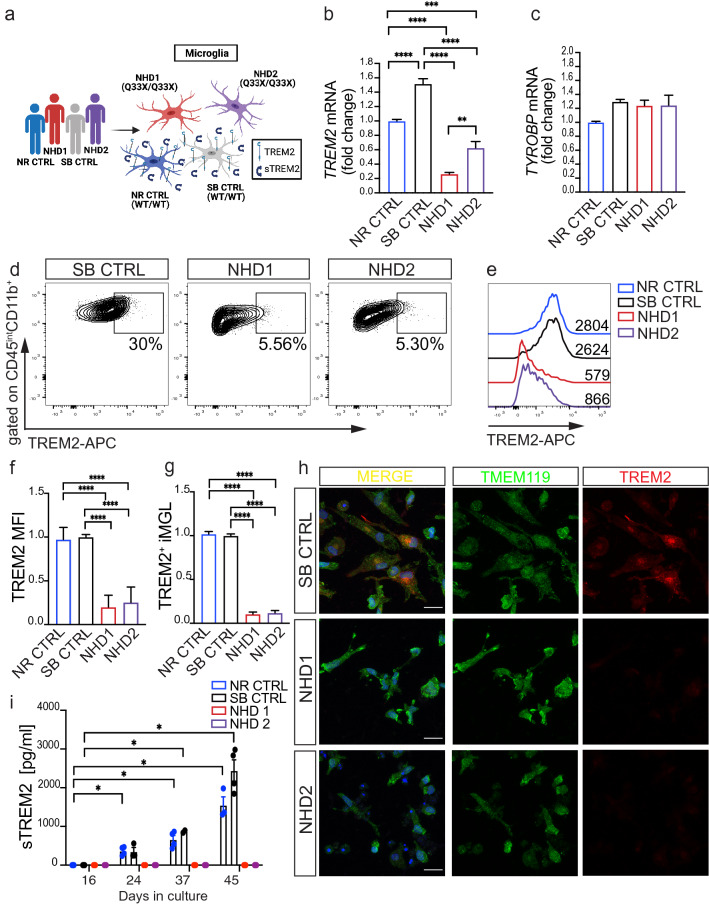
*TREM2* p.Q33X mutation abolishes cell surface expression of TREM2 receptor on iMGLs and abrogates sTREM2 production. **a** Diagram representing patient-derived iMGLs from NR CTRL, SB CTRL, NHD1 and NHD2 lines. **b** and **c** RT-qPCR of *TREM2* and *TYROBP* mRNA in NR CTRL, SB CTRL, NHD1 and NHD2 iMGLs. ***P < 0.001, ****P < 0.0001, One-way ANOVA with Tukey’s post hoc test. Data are pooled from two independent experiments. **d-g** FACS analysis of TREM2 receptor expression at iMGL cell surface. **d** Representative dot plots showing the percentage of TREM2^+^ cells in the CD45^int^ CD11b^+^ population. **e** Representative histogram of TREM2 mean fluorescent intensity (MFI) in SB CTRL, NHD1 and NHD2 iMGLs. **f** Quantification of TREM2 MFI and **g** TREM2^+^ iMGLs in NR CTRL, SB CTRL, NHD1 and NHD2 iMGLs. ****P < 0.0001, One-way ANOVA with Tukey’s post hoc test. Data are pooled from four independent experiments. **h** Representative confocal images of iMGLs from SB CTRL, NHD1 and NHD2 stained for TMEM119 (green), TREM2 (red) and DAPI (blue). Scale bar, 10 μm. **i** Media from NR CTRL, SB CTRL, NHD1 and NHD2 iMGLs were collected during iMGLs differentiation and sTREM2 was measured by ELISA. *P < 0.05, Two-way ANOVA with Dunnett’s post hoc test. Data are pooled from two independent experiments. Data shown are mean ± SEM

We next sought to determine the impact of *TREM2* p.Q33X on microglia phenotypes. iMGLs were differentiated from the NHD cases (NHD1 and NHD2) and the healthy sibling control (SB CTRL) (Fig. 2a). Transcriptomic analyses revealed that microglia-enriched genes are similarly expressed in the SB CTRL, NHD1 and NHD2 iMGLs (Supplementary Fig. 2f, online resource). Thus, NHD and control iMGLs maintain a similar propensity to form fully differentiated microglia-like cells. As expected, *TREM2* mRNA transcripts were significantly reduced in NHD1 and NHD2 iMGLs compared to NR and SB CTRL (p = 4.28 × 10^–41^, Supplementary Fig. 2f and Fig. 2b, online resource), while *TYROBP* mRNA levels were comparable across iMGLs (Fig. 2c). TREM2 protein was present at the membrane and in the intracellular compartment in NR and SB CTRL and in NR CTRL2 iMGLs but was absent in NHD1, NHD2 and NHD3 iMGLs by flow cytometry (FACS) (Fig. 2d–g and Supplementary 2 g-k, online resource), immunofluorescence (Fig. 2h and Supplementary Fig. 2b, online resource), and immunoblotting (Supplementary Fig. 2 l, online resource). Additionally, NR CTR, SB CTRL and NR CTRL2 iMGLs produced robust sTREM2 levels in culture medium, while sTREM2 was absent in medium from NHD1, NHD2 and NHD3 iMGLs (Fig. 2i and Supplementary Fig. 2 l, online resource). Interestingly, HET1 and HET2 iMGLs from the second NHD family showed a mixed phenotype. Membrane and soluble TREM2 levels in HET1 were comparable to NR CTRL2 iMGLs, while in HET2 the receptor and its soluble form were undetectable, similar to NHD3 iMGLs (Supplementary Fig. 2i–l, online resource). Together, these findings illustrate that the *TREM2* p.Q33X mutation negatively impacts the production of membrane and soluble TREM2 in iMGLs.

Beyond direct effects on TREM2*,* the *CCL3* gene (macrophage inflammatory protein-1 a, MIP-1a) was significantly reduced in NHD iMGLs compared to SB CTRL (p = 2.42 × 10^–24^, Supplementary Fig. 2f, online resource). *CCL3* is a neutrophil chemoattractant and a direct stimulator of osteoclastogenesis, a process that together with bone remodeling is controlled by TREM2/DAP12 signaling and is altered in NHD patients [10, 63, 70, 75]. We also observed a higher number of annexin-V^+^ (AnxV) propidium iodide (PI)^+^ cells in both NHD1 and NHD2 iMGLs compared to controls (Supplementary Fig. 2n and o, online resource), with NHD2 showing more cell death than NHD1 (Supplementary Fig. 2o, online resource). This finding is consistent with a role of TREM2 in sustaining microglial survival [67, 76, 102] and suggests that we can capture disease-relevant signatures in a dish.

### Downregulation of antigen presentation and immune activation pathways in NHD iMGLs and macrophages

**Fig. 3:**
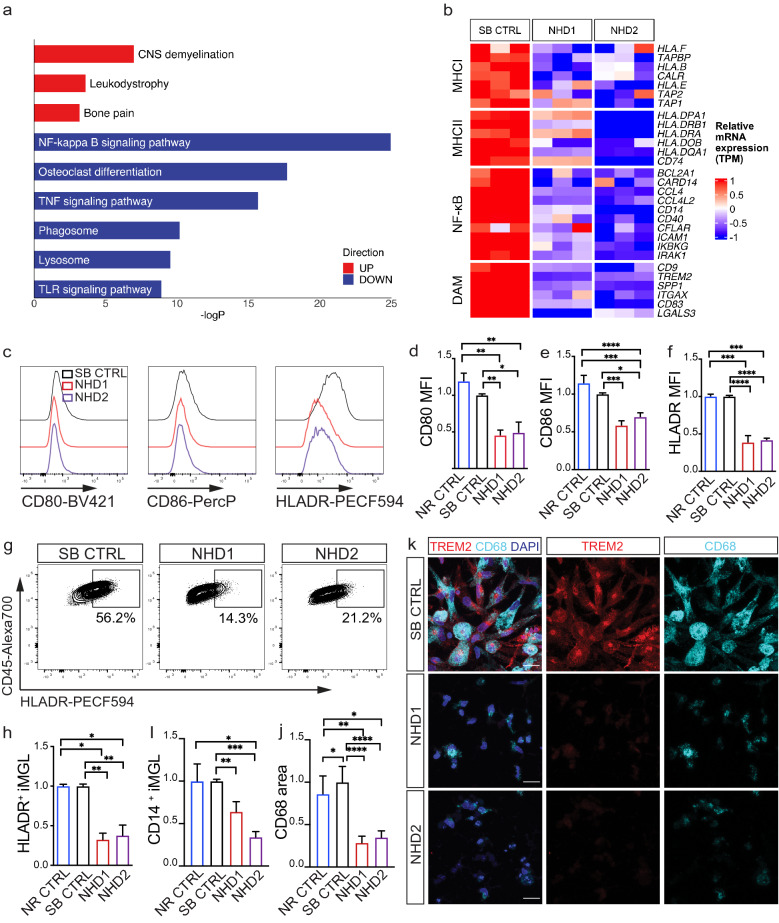
Defective immune activation in NHD iMGLs. **a** Pathway analysis of protein coding differentially expressed genes (DEG) in NHD versus SB CTRL iMGLs (SB CTRL, NHD1, NHD2; n = 3 replicates/line; EnrichR; DEG are defined by FDR < 0.05, DEGs in Supplementary Table 2, online resource)**.** **b** Heatmap of DEGs belonging to MHC I, MHC II, NF-κB and DAM pathways in iMGLs. **c** Representative histograms. **d-f** relative quantification performed by FACS showing cell surface **d** CD80, **e** CD86 and **f** HLA-DR MFIs in NR CTRL, SB CTRL, NHD1 and NHD2 iMGLs. **g** Representative dot plots showing the percentage (%) HLA-DR^+^ cells in SB CTRL, NHD1 and NHD2 iMGLs. **h** Quantification of % HLA-DR^+^ and i) CD14^+^ cells in NR CTRL, SB CTRL, NHD1 and NHD2 iMGLs. *P < 0.5, **P < 0.01, ***P < 0.001, ****P < 0.0001, One-way ANOVA with Tukey’s post hoc test. Data are pooled from four independent experiments. **j** Quantification of CD68 area in NR CTRL, SB CTRL, NHD1 and NHD2 iMGLs. **k** Representative confocal images of iMGLs stained for CD68 (cyan), TREM2 (red) and DAPI (blue). *P < 0.05, **P < 0.01, ****P < 0.0001, One-way ANOVA with Kruskal Wallis test and Dunn’s multiple comparison test. Data are pooled from two independent experiments. Data shown are mean ± SEM

To understand the broad impact of *TREM2* p.Q33X on microglia function, we performed transcriptomic analyses in iMGLs generated from SB CTRL, NHD1 and NHD2. We identified 3,353 differentially expressed genes when comparing NHD iMGLs with SB CTRL (using a cutoff of ± 1 log fold change and an FDR < 0.05; Supplementary Table 2 and Supplementary Fig. 3a and 3b, online resource). We found that the genes upregulated in NHD iMGLs (1,950 genes) belong to pathways associated with CNS demyelination (p = 9.36 × 10^–4^), leukodystrophies (p = 0.02), and bone pain (p = 0.04) (Fig. 3a and Supplementary Table 3, online resource). Among the genes downregulated in NHD iMGLs (1,816 genes), we observed an enrichment in genes involved in NF-κB signaling (p = 1.46 × 10^–11^), osteoclast differentiation (p = 2.06 × 10^–8^), tumor necrosis factor (TNF) signaling (p = 1.58 × 10^–7^), phagosome (p = 3.76 × 10^–5^), lysosome (p = 7.31 × 10^–5^), and Toll-like receptor (TLR) signaling (p = 1.37 × 10^–4^) (Fig. 3a and Supplementary Table 3, online resource). Genes involved in human leukocyte antigen (HLA) presentation and belonging to the major histocompatibility complex (MHC) class I and MHC class II clusters were also significantly reduced in NHD iMGLs compared to SB CTRL: *HLA.F, TAPBP, HLA.B, CALR, HLA.E, TAP2, TAP1, HLA.DPA1, HLA.DRB1, HLA.DRA, HLA.DOB, HLA.DQA1, CD74* (Fig. 3b). A defect in immune activation in NHD iMGLs was confirmed at the protein level by flow cytometry in both families, with a significant reduction of the costimulatory molecules CD80 and CD86 expressed as mean fluorescence intensity (MFI) (Fig. 3c–e and Supplementary 4a and b, online resource), HLA-DR expressed as MFI (Fig. 3c, f) and cell percentage (Fig. 3g and h, Supplementary Fig. 4c and d, online resource), and CD14 (Fig. 3i) at the cell surface of NHD1, NHD2 and NHD3 iMGLs compared to controls. HET2 iMGLs were comparable to NR CTRL2 iMGLs (Supplementary Fig. 4a–d, online resource), while HET1 displayed increased levels of CD80 and CD86 molecules compared to NR CTRL2 iMGLs (Supplementary Fig. 4a and b, online resource). We observed reduced expression of the phagocytic receptor MERTK [29, 92] in NHD1, NHD2 and NHD3 compared to NR CTRL, SB CTRL, NR CTRL2 iMGLs (Supplementary Fig. 4e–h, online resource), which is in line with phagocytic defects observed in *Trem2* knockout mouse and *TREM2* knockout human cells models [67]. MERTK^+^ iMGLs were comparable between HET1 and NR CTRL2 iMGLs, while MERTK was lower in HET2 iMGLs, thus further reflecting the phenotypic overlap between HET2 and NHD3 iMGLs (Supplementary Fig. 4 h, online resource). A decrease in immunostaining of the phagocytic marker CD68 in NHD1, NHD2 and NHD3 iMGLs was also observed, consistent with an overall decrease in activation (Fig. 3j and k, Supplementary Fig. 4i first NHD family; Supplementary Fig. 4j and k, second NHD family, online resource). Thus, under basal conditions, NHD iMGLs express a molecular and cellular phenotype consistent with a hypo-reactive phenotype.

To determine whether the effects of *TREM2* p.Q33X are conserved across myeloid cell populations, we compared transcriptomic data generated from PBMC-derived macrophages from the three siblings along with the iMGLs described above (Supplementary Fig. 3a and Fig. 1d, online resource). NHD macrophages produced a significant dysregulation of genes (2266 genes) enriched in pathways associated with proteasome (p = 1.58 × 10^–11^), antigen processing and presentation (p = 7.52 × 10^–6^), and steroid biosynthesis (p = 2.27 × 10^–7^) compared to SB CTRL (Supplementary Fig. 3c–d; Supplementary Table 4, online resource). We identified 345 genes that were commonly differentially expressed between NHD and SB CTRL in iMGLs and macrophages (Supplementary Fig. 3e–f, online resource). These commonly differentially expressed genes were enriched in pathways involved in phagocytosis (p = 3.38 × 10^–4^), response to interferon gamma (IFNγ) (p = 1.40 × 10^–3^), antigen processing and presentation (p = 1.23 × 10^–2^), regulation of apoptotic signaling pathways (p = 3.94 × 10^–3^), and lipid catabolic process (p = 9.36 × 10^–3^) (Supplementary Fig. 3 g, online resource). Thus, *TREM2* p.Q33X results in the dysregulation of phagocytic, activation, and lipid metabolic pathways across the myeloid lineage.

*Trem2* expression is required for the transition to disease associated microglia (DAM) in mouse models of neurodegeneration [46, 55] and in human microglia transplanted in a mouse model of amyloid accumulation [67]. Thus, we sought to determine the impact of *TREM2* p.Q33X on genes enriched in the DAM signature in NHD and SB CTRL iMGLs. We found that the DAM genes were reduced in NHD iMGLs (Fig. 3b), consistent with the requirement of *Trem2* for these signatures (Fig. 3b) [46]. *Trem2* deficiency is associated with an overactive response to bacterial lipopolysaccharide (LPS)-induced inflammation in mouse bone marrow-derived macrophages [61, 100], but studies using human iMGLs from NHD mutation carriers have generated conflicting observations [12, 32]. We investigated the effect of LPS, a well-characterized activator of TLR4 and a binding partner of TREM2 [19] in control and NHD iMGLs. We found that LPS treatment led to significantly elevated levels of HLA-DR, CD80, and CD86 in NHD1 and NHD2 iMGLs compared to SB CTRL iMGLs, suggesting an exaggerated response (Supplementary Fig. 4 l–n, online resource). Thus, NHD iMGLs exhibit hypoactivation under basal conditions and hyperactivation in response to inflammatory stimuli.

### Lysosomal defects in NHD iMGL

**Fig. 4:**
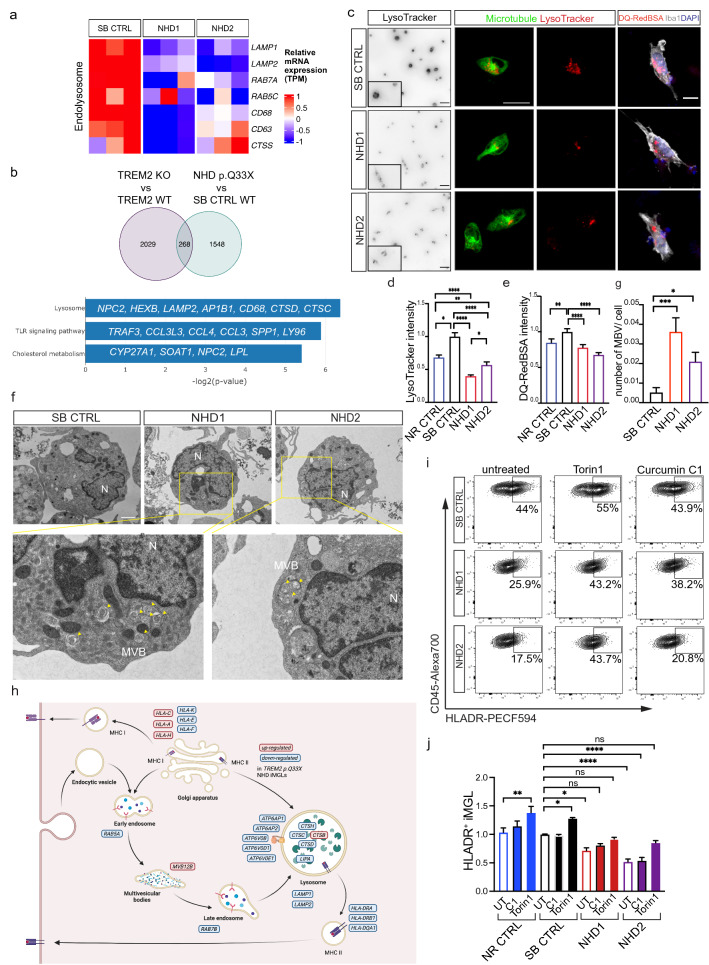
Lysosomal defects and multivesicular body accumulation in NHD iMGLs. **a** Heatmap of differentially expressed endo-lysosomal genes *LAMP1, LAMP2, RAB7A, CTSS, CD63, CD68, RAB5C* in iMGLs from NHD versus SB CTRL. **b** Venn diagram of downregulated genes shared between CRISPR/Cas9-engineered *TREM2* knockout (KO) *versus* WT published in [67] and NHD *versus* SB CTRL (p < 0.05; common DEGs = 268) and pathway analysis of the 268 DEGs in common between *TREM2* KO *versus* WT and NHD *versus* SB CTRL iMGLs. **c** Left panel, representative fluorescent images (in black and white) of iMGLs stained with LysoTracker Red. Insets represent enlarged images of cells. Scale bar, 10 μm. Middle panel, confocal images of LysoTracker Red (red) and microtubule (ViaFluor® 488, green) staining in iMGLs. Scale bar, 20 μm. Right panel, confocal images of iMGLs stained with DQ-Red-BSA (red), Iba1 (grey) and DAPI (blue). Scale bar 10 μm. **d-e** Quantification of **d** LysoTracker and **e** DQ-Red-BSA intensity in NR CTRL, SB CTRL, NHD1 and NHD2 iMGLs; CTCF values are normalized to SB CTRL. LysoTracker: NR CTRL n = 127 cells; SB CTRL = 156 cells, NHD1 n = 168 cells; NHD2 n = 107 cells. DQ-Red-BSA: NR CTRL n = 131 cells; SB CTRL = 141 cells, NHD1 n = 177 cells; NHD2 n = 145 cells. *P < 0.05, **P < 0.01, ****P < 0.0001, One-way ANOVA with Kruskal Wallis test and Dunn’s multiple comparison test. Data are pooled from two independent experiments. **f** Representative electron microscopy images of SB CTRL, NHD1 and NHD2 iMGLs and higher magnification. Undigested material (lamellar structures) is detected within multivesicular bodies (MVBs) in NHD1 and NHD2 iMGLs (yellow arrowheads). N, nucleus. Scale bar, 2 μm (top) and 500 nm (bottom). **g** Quantification of the number of late endosome/MVBs with inclusion bodies normalized per cell area in SB CTRL, NHD1 and NHD2 iMGLs. SB CTRL n = 18 cells; NHD1 n = 18 cells; NHD2 n = 17 cells. *P < 0.05, ***P < 0.001, One-way ANOVA with Kruskal Wallis test and Dunn’s multiple comparison test. h) Diagram representing the upregulated (red) and downregulated (blue) genes in NHD iMGLs compared to SB CTRL belonging to lysosomal and phagocytic pathways. Adapted from BioRender (2022). Endocytic Pathway Comparison (Layout). Retrieved from https://app.biorender.com/biorender-templates/t-61fd5292e07d3d00a5bd4c21-endocytic pathway-comparison-layout. **i** Representative dot plots showing the % of HLA-DR^+^ iMGLs in SB CTRL, NHD1 and NHD2 iMGLs untreated or pre-treated with Torin1 (250 nM) or with the curcumin analog C1 (1 μM). **j** Quantification of % of HLA-DR^ +  ^iMGLs. *P < 0.05, **P < 0.01, ****P < 0.0001. One-way ANOVA with Holm-Šídák's post hoc test. Data are pooled from two independent experiments. Data shown are mean ± SEM

*Trem2* deficient mice fail to upregulate genes involved in lysosomal and degradative pathways during demyelination [72, 101], suggesting a role for TREM2 in endolysosomal function. However, the impact of *TREM2* mutations on endolysosomal function has not been fully explored. We found that genes involved in endolysosomal pathways (*LAMP1*, *LAMP2*, *RAB7A*, *CTSS*, *CD63*, *CD68*, and *RAB5C*) were significantly downregulated in NHD compared with SB CTRL iMGLs (Fig. 3a and Fig. 4a). In order to determine whether these gene signatures are driven by the loss of function effect of the *TREM2* p.Q33X, we examined transcriptomic data from *TREM2* WT and CRISPR/Cas9-engineered *TREM2* knockout (KO) iMGLs [67]. The absence of *TREM2* led to the reduction of 2,297 genes in the iMGLs isogenic pairs compared with the 1816 genes downregulated in NHD versus SB CTRL iMGLs, of which 268 genes were shared (Fig. 4b and Supplementary Table 5, online resource). Pathway analysis of the 268 commonly downregulated genes revealed an enrichment in TLRs (*TRAF3, CCL3L3, CCL4, CCL3, SPP1, LY96*); lysosome (*NPC2, HEXB, LAMP2, AP1B1, CD68, CTSD, CTSC*); and cholesterol (*CYP27A1, SOAT1, NCP2, LPL*; Fig. 4b). Thus, loss of TREM2 function (by gene mutation or deletion) drives altered immune response, lysosome function and lipid metabolism.

To determine whether the impact of *TREM2* p.Q33X on lysosomal gene expression leads to functional defects in lysosomal machinery, we labeled acidic organelles in NHD and CTRL iMGLs using the pH-sensitive dye, LysoTracker red DND-99 (Fig. 4c; Supplementary Fig. 5a-b, top panel, online resource). LysoTracker fluorescence was significantly reduced in NHD1, NHD2 and NHD3 iMGLs compared to NR CTRL, SB CTRL and NR CTRL2 iMGLs (Fig. 4c and d; Supplementary Fig. 5a–c, online resource), indicating defective vesicle acidification. To determine whether *TREM2* p.Q33X also affects proteolytic degradation, we stained NHD and control iMGLs with DQ-Red BSA, which captures cargo delivery and degradation [65]. DQ-Red BSA intensity was significantly reduced in NHD1, NHD2 and NHD3 compared to NR CTRL, SB CTRL and NR CTRL2 iMGLs, suggesting a defect in uptake or breakdown of the substrate (Fig. 4c right, and e; Supplementary Fig. 5b bottom panel and d, online resource). DQ-Red BSA intensity in HET1 and HET2 iMGLs were comparable to NR CTRL2 iMGLs. However, HET2 iMGLs displayed reduced by Lysotracker intensity compared to NR CTRL2 iMGLs (Supplementary Fig. 5c–d, online resource). Additionally, using electron microscopy, we detected an accumulation of unprocessed material within MVBs [35] in NHD1 and NHD2 iMGLs compared to the SB CTRL iMGLs (Fig. 4f and g). These findings suggest that *TREM2* p.Q33X, likely via TREM2 functional deficiency, leads to defects in lysosomal function that result in the accumulation of undegraded material in MVBs (Fig. 4h).

Proper assembly and recycling of MHC class II molecules to the cell membrane requires the endolysosomal system [17, 30]. As such, our observation of reduced HLA-DR expression in NHD iMGLs may be a consequence of lysosomal dysfunction. In addition to the core lysosomal genes that were downregulated in NHD iMGLs, we observed a significant reduction of *TFEB* (p = 1.1 × 10^–2^) (Supplementary Table 2, online resource). *TFEB* is the master regulator of lysosomal biogenesis and positively controls autophagosome formation and autophagosome-lysosome fusion [84, 90, 93]. To determine whether restoring lysosomal function could rescue key defects in NHD iMGLs, we tested the impact of compounds that activate TFEB via mTOR-dependent (Torin 1) and -independent (curcumin C1) pathways. Pre-treatment with Torin 1 fully rescued HLA-DR expression in NHD1, NHD2 and NHD3 iMGLs to levels comparable to control iMGLs (Fig. 4i and j; Supplementary Fig. 5e–g, online resource). Torin 1 treatment also increased HLA-DR levels in NR CTRL, SB CTRL and NR CTRL2 (Fig. 4j; Supplementary Fig. 5 g, online resource). Curcumin C1 treatment did not affect HLA-DR expression in control iMGLs but was effective in restoring HLA-DR expression in NHD1 iMGLs only (Fig. 4i and j). This finding suggests that mTOR-dependent activation of TFEB may restore defective lysosomal pathways in iMGLs independent of TREM2 expression. Thus, rescuing lysosomal abnormalities in NHD iMGLs may have broader restorative effects on microglia function.

### Lipid metabolism is reduced in NHD iMGLs

**Fig. 5:**
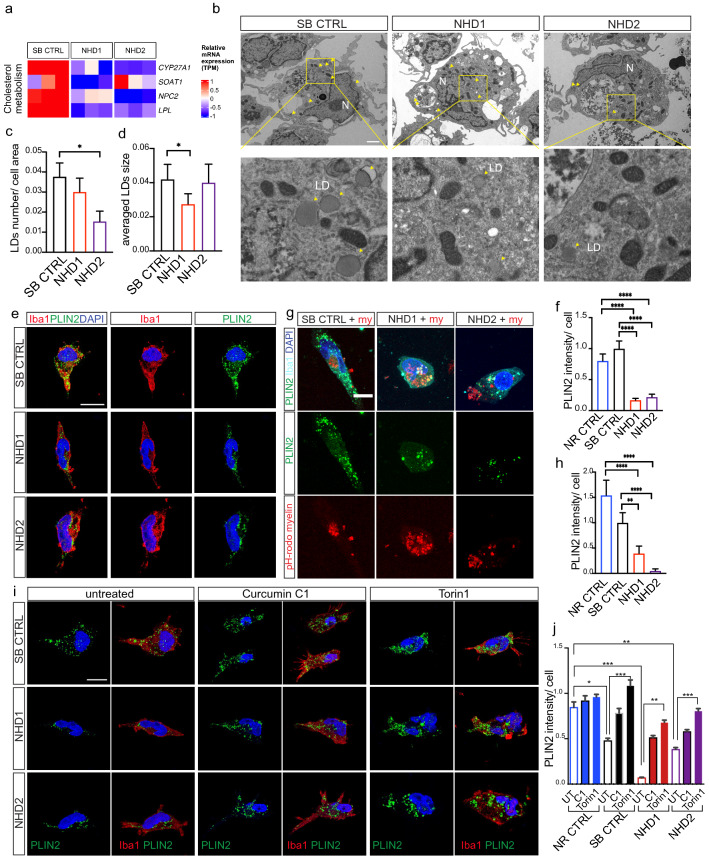
NHD iMGLs display reduced number of lipid droplets. **a** Heatmap of cholesterol pathway genes *CYP27A1, SOAT1, NCP2, LPL* in iMGLs from NHD versus SB CTRL. **b** Representative electron microscopy images of SB CTRL, NHD1 and NHD2 iMGLs showing lipid droplets (LD) (yellow arrowheads). At the bottom are enlarged images of the enclosed area. N, nucleus. Scale bar, 2 μm. **c** Quantification of the number of lipid droplets per cell area in SB CTRL, NHD1 and NHD2 iMGLs. **d** Quantification of the average of lipid droplets size in SB CTRL; NHD1 and NHD2 iMGLs. SB CTRL n = 18 cells; NHD1 n = 18 cells; NHD2 n = 17 cells. *P < 0.05, One-way ANOVA with Tukey’s post hoc test. **e** Representative confocal images of Perilipin2 (PLIN2) (green) in Iba-1^+^ iMGLs (red), DAPI (blue). Scale bar, 10 μm. **f** Quantification of PLIN2 intensity. NR CTRL n = 77 cells; SB CTRL = 101 cells, NHD1 n = 97 cells; NHD2 n = 69 cells. ****P < 0.0001, One-way ANOVA with Kruskal Wallis test and Dunn’s multiple comparison test. Data are pooled from three independent experiments. **g** Representative confocal images of PLIN2 (green) in Iba-1^+^ iMGLs (cyan), DAPI (blue), treated with pH-rodo human myelin (my; red) for 24 h. Scale bar, 10 μm. **h** PLIN2 intensity quantification in iMGLs treated with pH-rodo human myelin. NR CTRL n = 37 cells; SB CTRL = 39 cells, NHD1 n = 47 cells; NHD2 n = 39 cells. **P < 0.01, ****P < 0.0001, One-way ANOVA with Kruskal Wallis test and Dunn’s multiple comparison test. Data are pooled from two independent experiments. **i** Representative confocal images of Perilipin2 (PLIN2) (green) in Iba-1^+  ^iMGLs (red), DAPI (blue) in SB CTRL, NHD1 and NHD2 iMGLs untreated or pre-treated with Torin1 (250 nM) or with the curcumin C1 (1 μM). Scale bar, 10 μm. **j** Quantification of PLIN2 intensity. NR CTRL n = 77 cells; SB CTRL = 101 cells, NHD1 n = 97 cells; NHD2 n = 69 cells. *P < 0.05, **P < 0.01, ***P < 0.001, One-way ANOVA with Kruskal Wallis test and Dunn’s multiple comparison test. Data shown are mean ± SEM

Lysosomal pathways play a critical role in processing and sorting exogenous and endogenous lipids [99]. *Trem2* is required for lipid droplet biogenesis in a toxin-induced model of CNS demyelination [34], and *Trem2*–deficient mice exhibit defects in lipid droplet formation due to excessive cholesterol [34]. *TREM2* p.Q33X iMGLs resulted in downregulation of cholesterol genes (*CYP27A1, SOAT1, NCP2, LPL*) (Fig. 4b and Fig. 5a). Thus, we sought to investigate whether NHD iMGLs display defects in lipid droplet biogenesis. Electron microscopy revealed a significant decrease of lipid droplet number in NHD2 compared to SB CTRL iMGLs (Fig. 5b and c). NHD1 showed a reduction of lipid droplet size compared to SB CTRL iMGLs without a significant change in droplet numbers (Fig. 5b–d). Additionally, we observed a significant reduction in the intensity of Perlipin2 (PLIN2) expression, a protein that coats intracellular lipid droplets, in NHD1, NHD2 and NHD3 iMGLs compared to NR CTRL, SB CTRL and NR CTRL2 iMGLs (Fig. 5e and f; Supplementary Fig. 5 h–j, online resource). Staining iMGLs with the fluorescent neutral lipid dye Bodipy, which illuminates neutral lipids, revealed a decrease in Bodipy intensity in NHD1 and NHD2 iMGLs compared to SB CTRL (Supplementary Fig. 5 k–l, online resource). Myelin debris are actively phagocytosed by microglia during demyelination to promote clearance and repair of the brain, with TREM2 receptor playing a pivotal role in this process [15, 88]. We observed that NR CTRL, SB CTRL, NHD1 and NHD2 iMGLs phagocytosed pHrodo-conjugated human myelin, as shown by confocal acquisition (Supplementary Fig. 5 m, online resource). Thus, NHD iMGLs were able to engulf cholesterol-enriched substrates such as myelin and efficiently transport them to the intracellular lysosomal compartment. To investigate whether cholesterol-rich myelin has an effect on lipid droplet content in NHD iMGL, we next treated control and NHD iMGLs with human myelin for 24 h. Even in the presence of myelin, we still detected a reduced amount of PLIN2^+^ staining in NHD1 and NHD2 iMGL compared to NR and SB CTRL (Fig. 5g and h). Pre-treatment with Torin 1 and Curcumin C1 fully rescued PLIN2 protein expression in NHD1 and NHD2 iMGLs to levels comparable to SB CTRL and NR CTRL (Fig. 5i and j; Supplementary Fig. 5n, online resource). Torin 1 and Curcumin C1 treatment also increased PLIN2 levels in NR CTRL and SB CTRL (Fig. 5j). Thus, rescuing lysosomal abnormalities in NHD iMGLs may have broader restorative effects on microglia function.

### Lysosomal and metabolic defects in NHD brain tissues

**Fig. 6:**
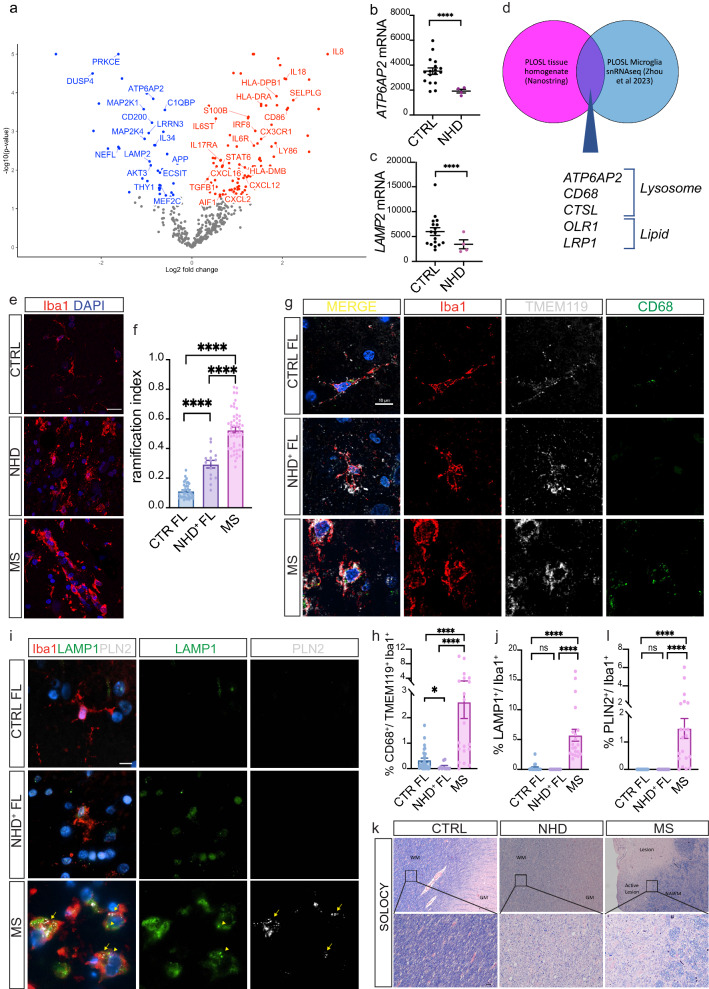
Post-mortem tissues from NHD patients display lysosomal and lipid metabolism defects. **a** NanoString analysis was performed on hippocampi from four NHD patients (one *TREM2 D134G*; one *TREM2* c.482 + 2 T > C; two *TYROBP* c.141delG) and 17 controls. Volcano plot of DEG between NHD and control brains (p < 0.05). Red: upregulated genes; blue: downregulated genes. **b** *ATP6AP2*, and **c** *LAMP2* mRNA (raw counts) from NanoString analysis of NHD versus control brains. ****P < 0.0001. **d** Venn diagram of DEGs shared between NanoString analysis of PLOS tissues homogenates (hippocampi) *versus* controls (p < 0.05) and the DEGs of PLOS (n = 3) *versus* controls (n = 11) in the microglia cluster from snRNA-seq dataset [108] (Supplementary Table 7, online resource). **e** Representative confocal images of Iba-1^+^ microglia (red) and DAPI (blue) staining of frontal lobe (FL) regions of a healthy control, one NHD patient (NHD1^+^ (FL)), and one multiple sclerosis (MS) patient (active lesion in the medulla). Scale bar, 20 μm. **f** Microglia morphometric analysis of FL regions of three healthy controls, one NHD1^+^ (FL), and two MS patients. The analysis was performed using a ramification index [RI = 4π × cell area/(cell perimeter)^2^] that captures microglia cell shape. FL: CTRL n = 38 cells; NHD1^+^ n = 15 cells; MS n = 50 cells. ****P < 0.0001, analysis was performed between two groups using an Unpaired T test. **g** Representative fluorescence images of Iba-1^+^ microglia (red), TMEM119 (gray), CD68 (green) and DAPI (blue) staining of FL regions of a healthy control, one NHD patient (NHD1^+^ (FL)), and one MS patients (active lesion in the medulla). Scale bar, 10 μm. **h** Quantification of CD68^+^ signal per TMEM119^+^ Iba1^+^ cell. FL: CTRL n = 28 cells; NHD1^+^ n = 10 cells; MS n = 20 cells. *P < 0.05, ****P < 0.0001, analysis was performed between two groups using a Mann–Whitney T test. **i** Representative images of Iba-1^+^ microglia (red), PLIN2 (grey), LAMP1 (green) and DAPI (blue) staining of FL regions of a healthy control, one NHD patient (NHD1^+^ (FL)), and one MS patients (active lesion in the medulla). Scale bar, 20 μm. **j** Quantification of LAMP1 signal per Iba1^+^ cell. LAMP1 intensity: FL: CTRL n = 17 cells; NHD1^+^ n = 10 cells; MS n = 20 cells. ****P < 0.0001, analysis was performed between two groups using a Mann–Whitney T test. **k** White matter (WM) and grey matter (GW) regions with no pathology in one control individual (CTRL), one NHD patient, and one MS patient (normal appearing white matter, NAWM; and active lesions in the white matter, MS ACTIVE) were stained with solochrome cyanine staining (SOLOCY). Original magnification: 4X and 20X; scale bar: 250 μm and 25 μm, respectively. **l** Quantification of PLIN2 signal per Iba-1^+^ cell. PLIN2 intensity: FL: CTRL n = 17 cells; NHD1^+^ n = 10 cells; MS n = 20 cells. ****P < 0.0001, analysis was performed between two groups using a Mann–Whitney T test

To determine the extent to which the NHD iMGLs phenotypes are represented in NHD patients, we examined brain tissues from NHD and healthy controls. First, we analyzed gene expression data by NanoString in hippocampi [54] from four NHD patients and 17 healthy controls (Supplementary Table 1, online resource). We identified 122 significantly upregulated genes and 46 downregulated genes among the 800 genes evaluated (p value < 0.05) (Fig. 6a and Supplementary Table 6, online resource). Genes involved in neuroplasticity, synaptic function (*C1QBP, APP*, *NEFL*, *LRRN3*, *MEF2C*, *THY1*), and neuron-microglia crosstalk (*CD200* and *IL34*) were downregulated in NHD brains (Fig. 6a and Supplementary Table 6, online resource). We also observed a significant increase in genes associated with microgliosis (*CX3CR1* and *AIF*) and astrogliosis (*S100B*). The genes significantly upregulated in NHD brains were enriched in pathways associated with inflammation and immune activation (Fig. 6a), such as inflammatory cytokines (*IL8, IL18, IL6R, TGFB1, IL17RA)*; antigen presentation molecules (*IRF8, CD86*, *HLA-DPB1, HLA-DRA*); and chemokines (*CXCL2, CXCL12, CXCL16, SELPLG*) (Supplementary Table 6, online resource). These findings illustrate that post-mortem brain tissues from NHD patients display a profound effect on immune cell activation and inflammation, which is consistent with the dysregulation of these pathways in iMGLs and with the exaggerated response to LPS. Among the downregulated genes in NHD brains, we observed genes enriched in pathways involved in the activation of mitogen-activated protein kinase (MAPK)-mediated cascade and NF-κB (*PRKCE, DUSP4, MAP2K1, MAP2K, AKT3, ECSIT)* [85, 105]. Strikingly, *ATP6AP2* and *LAMP2,* which are involved in lysosomal acidification and lysosomal protein degradation [24, 48], were significantly downregulated in NHD brains compared to controls (p = 1.44 × 10^–4^ and p = 5.95 × 10^–3^, respectively) (Fig. 6b and c). NanoString analysis was performed on tissue homogenates from the hippocampus; therefore, dysregulated genes could reflect changes in brain cell types beyond microglia. We compared the dysregulated genes from our NanoString analyses with genes identified in a recent single-nucleus RNA-seq (snRNA-seq) dataset composed of postmortem specimens from 3 individuals affected by NHD and carrying homozygous mutations in the *TYROBP* gene [108]. Focusing on the microglia snRNA-seq cluster, we found that genes involved in lysosomal function such as *ATP6AP2*, *CD68* and *CTSL* were downregulated in microglia (Supplementary Table 7 and Fig. 6d, online resource). *OLR1* and *LRP1*, which are involved in lipid metabolism, were significantly downregulated in the microglia cluster. Additionally, genes involved in microglia activation were also downregulated in the snRNA-seq microglial cluster (*HLA-DPB1*, *HLA-DRA*, *CD86*, *HLA-DPA1*) thus corroborating our observation of a defective activation in iMGLs. These findings strongly support our observations of lysosomal, lipid metabolism and activation defects in NHD iMGLs (Fig. 4). Thus, we showed that key phenotypic features observed in *TREM2* p.Q33X iMGLs, such as defects in immune activation and lysosomal dysfunction, are conserved in human brains from NHD patients.

We next examined microglia morphology in tissues from two NHD patients, three individuals with no evidence of CNS pathology, and two patients affected by multiple sclerosis (MS), a prototypic neurological disease characterized by myelin loss (Supplementary Table 1, online resource). NHD is an exceedingly rare disease, and tissue from NHD patient brains that have come to autopsy are limited. Given these limitations, we analyzed the frontal lobe of one NHD patient (named NHD^+^) and the basal ganglia of a second NHD patient (named NHD^+ +^). In the healthy CNS, microglia are retained in a surveillance state and usually display a highly ramified morphology, while in pathological condition microglial cells can become ‘reactive’ and acquire an amoeboid shape, with a range of intermediate activation states in between [58]. By analyzing the ramification index (a morphological measure of microglia activation), we observed that Iba-1^+^ cells from NHD^+^ and NHD^++^ patients exhibited a dystrophic appearance and decreased ramification compared to controls, which corresponds to a reactive status (Fig. 6e and f; Supplementary Fig. 6a, online resource). Microglia in active lesions from MS patients were significantly more rounded and less ramified compared to NHD and CTRL patients, and thus highly reactive (Fig. 6e and f; Supplementary Fig. 6a, online resource). This observation confirms that microglia from NHD patients are aberrantly activated and dystrophic, which is distinct from reactive microglia in MS lesions.

To investigate whether lysosomal markers were altered in NHD brains, we quantified CD68 intensity in TMEM119^+^Iba-1^+^ microglia from control and NHD^+^ tissues and in tissues from one MS patient (Fig. 6g). CD68 was significantly reduced in the frontal lobe of NHD^+^ compared to CTRL, while it was significantly increased in MS tissues (Fig. 6g and h). The lysosomal membrane glycoprotein LAMP1 detected in Iba-1^+^ cells was not changed in NHD^+^ compared to controls in the frontal lobe, while it was upregulated in MS tissues (Fig. 6i and j). Notably, LAMP1 intensity detected in the basal ganglia of NHD^++^ was significantly higher than CTRL (Supplementary Fig. 6b-c, online resource). These findings highlight the extent to which brain regions could be impacted to varying degrees during pathophysiology. Reduced CD68 staining observed in the frontal lobe of the NHD^+^ patient is consistent with the dysfunction in the lysosomal machinery and accumulation of degradative vesicles described in NHD iMGLs (Fig. 4). Strikingly, tissues from NHD patients exhibited areas of diffuse demyelination compared to control patients, which was similar to white matter (WM) active lesions detected in the MS patients (Fig. 6k). However, while myelin debris were largely cleared in the MS lesion, in NHD, myelin debris remained in the tissue, as shown by a diffuse blue staining (Fig. 6k). The defect in myelin debris clearance may be due to the reduced degradative capacity of the NHD microglia owing to the observed lysosomal defects. We next stained for PLIN2 in control, MS and NHD brain tissues (Fig. 6i; Supplementary Fig. 6b, online resource). There was a significant increase in PLIN2 signal in Iba-1^+^ cells in the MS active lesions (Fig. 6l). Despite microglial activation and the inflammation observed in NHD tissues, PLIN2 staining in both frontal lobe and basal ganglia of NHD^+^ and NHD^++^ was significantly lower than the signal detected in MS patients and was comparable to controls (Fig. 6i and l; Supplementary Fig. 6d, online resource). This result is consistent with the findings in NHD iMGLs showing reduced lipid droplets, PLIN2, and Bodipy staining in the presence and absence of myelin (Fig. 5). Thus, while demyelination and lipid metabolism dysregulation are both involved in NHD and MS [13], the cellular phenotypes exist on a spectrum, which may reflect different pathological mechanisms driving these clinically distinct phenotypes.

## Discussion

Here, by combining transcriptomic and functional analyses in control and NHD iMGLs and brain tissues from NHD patients, we further improved our understanding of the mechanisms underlying this rare leukodystrophy. We showed that iMGLs derived from patients affected by NHD and carrying the *TREM2* p.Q33X mutation display dysregulation of lysosomal function, reduced lipid droplets, and downregulation of cholesterol genes. The defective activation and downregulation of HLA-DR molecules together with the reduction in the lipid droplets protein PLIN2 detected in NHD iMGLs were rescued by enhancing lysosomal biogenesis through mTOR-dependent and independent pathways. Alteration in the same pathways were also observed in post-mortem brain tissues from NHD patients, closely recapitulating in vivo the phenotype observed in iMGLs in vitro.

Thus far, murine models are not able to recapitulate the key phenotypic features observed in patients affected by NHD. This has become an increasingly pressing challenge as rare variants in *TREM2* have been identified as risk factors in Alzheimer’s disease and frontotemporal dementia [9, 37, 38, 42, 54]. Stem cell technologies have allowed us to obtain human cells from clinically defined patients, allowing for in-depth phenotypic analyses in exceedingly rare diseases such as NHD. Therefore, leveraging iMGLs represents a unique modeling platform to define disease-relevant, patient-specific phenotypes in a dish to understand disease mechanisms and for eventual therapeutic target identification, validation and drug discovery pipelines.

Transcriptional and functional analyses highlight that NHD iMGLs in vitro are hyporeactive compared to iMGLs from healthy controls, showing a deficit in HLA-DR, CD80, and CD86 molecules expression. This phenotype is reminiscent of what has been described in iMGL-chimeric mouse models harboring *TREM2* deficient microglia [67]. Single cell RNAseq on iMGLs isolated from engrafted mice showed that *TREM2* KO iMGLs were shifted toward a more homeostatic profile even in wild-type mice, with a specific decrease of genes involved in the MHC-II and HLA presentation. Thus, the absence of TREM2 maintains iMGLs in a quiescent state, but this dormant state is released after LPS activation [67]. Conversely, in NHD and controls brain tissues, NanoString analysis revealed the upregulation of genes involved in immune cell recruitment and activation, inflammation, microgliosis and astrocytosis. Analysis of overlapping genes between the microglial cluster from snRNA-seq dataset in Zhou Y at el. [108] and our NanoString dataset revealed the downregulation of genes involved in lysosomal function, lipid metabolism and microglia activation. Upregulation of genes involved in inflammation and activation captured by NanoString analysis may be due to other brain cells (i.e. astrocytes, perivascular cells). The discrepancy between the inflammatory status observed in NHD tissues and the hyporeactive phenotype detected in iMGLs in culture, may be explained by the nature of the model system, where iMGLs capture function and transcriptome of cells isolated in culture, while post-mortem tissues are an end-stage snapshot resulting from the interaction of microglia with a much more complex cellular milieu after years of accumulating pathology. One possible scenario is that NHD microglia could be hypoactive early during the disease, and this abortive activation in NHD brains could lead later to a dystrophic and pro-inflammatory microglial phenotype. The hyperactivation observed in NHD iMGLs in vitro after LPS challenge indeed supports this possible scenario. To further support this hypothesis, in a model of acute CNS demyelination, lack of TREM2 was associated with defective microglia activation, while in chronic demyelination, TREM2 deficient microglia acquired a more pro-inflammatory and potentially neurotoxic phenotype [13].

Multiple groups have examined the impact of NHD mutations on cellular phenotypes in iPSC-iMGLs models. These efforts have focused on the NHD mutations *TREM2* p.T66M, p.W60C, p.Y38C, p.V126G [12, 18, 32, 50, 53, 73, 80]. Despite differences in the protocols adopted to generate human microglia, a common phenotype reported includes a reduction in TREM2 and sTREM2 levels, defects in phagocytosis of apoptotic bodies, and reduced survival in the mutant microglia. LPS-mediated cytokine secretion was comparable between control and *TREM2* missense mutations in one study [32], while another group came to the opposite conclusion [12]. Microglia-like cells from patients carrying *TREM2* p.T66M*,* in either homozygous and heterozygous state, or *TREM2* p.W50C mutations, responded to LPS stimulation and were phagocytically competent despite accumulation of an immature form of TREM2 that was not trafficked to the plasma membrane [12]. We observed common gene signatures shared by *TREM2* p.Q33X carriers and *TREM2* deficient iMGLs [67]. These findings suggest that some, but not all, of the observed defects are driven by *TREM2* loss of function. Our sequencing results and functional experiments also highlight the differences between NHD1 and NHD2 iMGLs. This may be due to differences in the clinical severity of disease, other genetic modifiers, and/or sex-based microglial effects. Also, NHD1 and NHD2 iMGLs cultures exhibited some cell death, which was more pronounced in NHD2 compared to NHD1 iMGLs. Even though this finding is consistent with prior literature, supporting the role of the TREM2 receptor in microglial survival, we acknowledge that cell death in NHD iMGLs could constitute a confounding factor. Further studies are required to address these observations. Thus, studies of *TREM2* mutations are essential for capturing the full complexity of disease.

We show that lysosomal dysfunction is a common phenotype shared across NHD iMGLs and NHD brains. NHD iMGLs exhibit reduced labelling of acidic organelles and proteolytic capacity of degradative vesicles along with an accumulation of undegraded material in multivesicular bodies. To this end, we hypothesize that the accumulation of unprocessed material within the MVBs observed in NHD iMGLs is a consequence of the lysosomal defects. Similarly, we observed lysosomal defects in NHD brains, as shown by a decrease in CD68 protein staining in the frontal lobe of a NHD patient and decreased transcription of genes implicated in lysosomal acidification (*ATP6AP2)* and chaperone mediated autophagy (*LAMP2*). LAMP1 protein expression, which marks degradative vesicles, was increased in the basal ganglia, implicating regional-specific differences in NHD pathology. The dysregulation of lysosomal genes and accumulation of lysosomal debris have been widely reported in lysosomal storage diseases [87]. Lysosomal dysfunction has broad consequences on cellular function and may affect core signaling events of the cell [5, 8]. In this regard, MHC class II molecules and recycling of peptides displayed by MHC class II molecules are assembled in the endo-lysosomal compartment [17, 30]. Thus, the reduction observed in MHC I and MHC II genes and proteins in NHD iMGLs might be explained by the deficient endo-lysosomal transport preventing the correct assembly and delivery of these molecules at the membrane. By targeting lysosomal and autophagic processes through TFEB activation in an mTOR-dependent or independent manner, we fully restored HLA-DR levels in NHD iMGLs. TFEB colocalizes with master growth regulator mTOR complex 1 (mTORC1) on the lysosomal membrane [94], and pharmacological inhibition of mTORC1, activates TFEB by promoting its nuclear translocation. TREM2 activates mTOR through DAP12 and/or DAP10, via recruitment of upstream mTOR activators such as PI3K and AKT. TREM2 sustains cell metabolism through rapamycin complex 1 and 2 (mTORC1 and mTORC2, respectively) signaling and defective mTOR activation in TREM2-deficient microglia is associated with a compensatory increase of autophagy [101]. This is the first evidence that the lysosomal compartment could be a critical hotspot impacted during the disease by the presence of TREM2 mutations in human microglia, largely responsible for NHD pathophysiology. These findings suggest that the endolysosomal degradative pathway could be a novel and attractive therapeutic target for this disease or others characterized by altered TREM2 function in microglia [26, 87].

*TREM2* p.Q33X iMGLs show downregulation of lipid metabolism at the gene network and protein level. We observe a reduced amount of lipid droplets in NHD iMGLs compared to controls, both in the presence or absence of myelin. Lipid droplets are dynamic subcellular organelles required for storage of neutral lipids such as glycero-lipids and cholesteryl esters, and have been recently described as essential to metabolically support immune responses, antigen cross-presentation, interferon (IFN) responses, and production of inflammatory mediators [74]. Alteration in myeloid cell activation status is connected to profound changes in lipid droplets numbers and composition. Lipid droplets have also been shown to accumulate in microglia during aging, being part of a characteristic microglial transcriptional signature called lipid-droplets-accumulating microglia (LDAM), which show defects in phagocytosis, release elevated levels of proinflammatory cytokines and reactive oxygen species [64]. Observations of reduced lipid droplet content in NHD iMGLs are consistent with work showing that, upon demyelinating injury, *Trem2*-deficient mice are unable to elicit the adaptive response to excess cholesterol exposure, form fewer lipid droplets than wild-type mice, and develop endoplasmic reticulum stress [34]. This supports the hypothesis that the TREM2 receptor is required for lipid droplets biogenesis and to buffer cholesterol-mediated toxicity in mouse and human microglia. Another possible interpretation of our findings, is that lysosomal dysfunction in NHD iMGLs would lead to an altered degradation of these lipidic structures [106]. Further, our finding that lipid droplets content in NHD iMGLs was rescued via mTOR-dependent pathways is consistent with prior evidence that mTORC1 inhibition stimulates lysosomal hydrolysis of phospholipids and results in an adaptive shift in the use of constituent fatty acids which promotes their storage in newly formed lipid droplets for energy production [40].

Finally, we observed the downregulation of genes involved in neuroplasticity, synaptic function and microglia-to-neuron cross-talk in NHD brains versus controls. Microglial survival is controlled by IL34 and colony-stimulating factor 1 (CSF1), through CSF1R signaling activation [23, 43]. Microglia, in turn, are critical for the regulation of neuronal activity and firing in a region-specific and microglia number-dependent manner [4]. This is highlighted by observations that NHD patients do not only display defects at the microglial level but instead present with neuronal phenotypes and dysfunction that might be microglial-dependent and/or independent. This area of study raises many new questions about the impact of *TREM2* mutations on neuronal function during neurodegeneration and highlights how studying NHD might help us also understand microglia-to-neuron cross-talk and the impact of *TREM2* mutations on this interaction. Overall, our results suggest that NHD might be considered a lysosomal-storage disease with white matter alterations as first proposed in reports based solely on NHD pathology [49] when the genetic cause of the disease was not known. Our study provides the first cellular and molecular evidence that lack of TREM2 in microglia leads to a defect in lysosomal function. A better understanding of how microglial lipid metabolism and lysosomal machinery are altered in NHD may provide new insights into mechanisms underlying NHD pathogenesis.

## Supplementary Information

Below is the link to the electronic supplementary material.

Supplementary Figure 1: Generation and characterization of iPSCs from NHD patients and non-carrier family member. a-b) Karyotyping of a) iPSCs from SB CTRL and two affected siblings (NHD1 and NHD2) and b) NR CTRL2, two heterozygous parents (HET1 and HET2) and one affected child (NHD3). c-d) iPSCs were stained for octamer-binding transcription factor (OCT4), SRY-Box Transcription Factor 2 (SOX2), Stage-specific embryonic antigen-4 (SSEA4) or Transcription-associated protein 1-60 (Tra-1-60) as indicated for c) SB CTRL, NHD1 and NHD2, Scale bar, 20 μm. d) NR CTRL2, HET1, HET2 and NHD3. Scale bar, 50 μm. e-f) Sanger sequencing of TREM2 p.Q33X mutation. Sequence chromatogram showing the C to T point mutation at position 97 (C97T) in exon 2 of the TREM2 gene. *, variant site. Characterization of unrelated control iPSCs (NR CTRL) line was reported in [45]. Supplementary Figure 2: Validation and characterization of fully differentiated iMGLs. a) Schematic of iMGLs differentiation protocol (see Methods for additional information). After 40 days in culture (starting from iPSCs) iMGLs are fully mature and used for analyses. b) Confocal images of iMGLs from healthy donors (NR CTRL) stained for TMEM119, P2RY12, Iba-11 and TREM2. Scale bar, 20 μm. c) sTREM2 detected by ELISA in the media of CTRL iMGLs during the differentiation process. ***P < 0.001, ****P < 0.0001, One-way ANOVA with Dunnett’s post hoc test. Data are pooled from two independent experiments. d) Representative dot plot showing CD11b and CD45 cell surface expression in fully mature iMGLs (blue) versus macrophages (MΦ; red) from control donors. e-f) RNAseq was performed on iMGLs (N=1 SB CTRL, N=2 NHD lines; n=3 replicates/line) and MΦ (N=1 SB CTRL, N=2 NHD lines; n=3 replicates/line) (See Supplementary Table 1 for donor details, online resource). e) 3D principal component analysis (PCA) of iMGLs (red) and MΦ (blue) using the 500 genes with the highest variance (TPM normalized counts). f) Heatmap showing homeostatic microglia signature genes highly expressed by iMGLs compared to MΦ in NHD versus SB CTRL. g) Representative dot plots showing the % of TREM2^+^ cells in NR CTRL (first NHD family). h) Diagram representing patient-derived iMGLs from NR CTRL2, HET1, HET2 and NHD3 lines. i-k) FACS analysis of TREM2 receptor expression at the surface of iMGLs for the second NHD family. i) Representative dot plots showing the % of TREM2^+^ cells in the CD45^int^ CD11b^+^ population. j) Quantification of TREM2^+^ cells in NR CTRL2, HET1, HET2 and NHD3 iMGLs. **P < 0.01, ****P < 0.0001, One-way ANOVA with Holm-Šídák’s post hoc test. Data are pooled from two independent experiments. k) Representative histogram of TREM2 mean fluorescent intensity (MFI) in NR CTRL2, HET1, HET2 and NHD3 iMGLs. l) Immunoblots for TREM2 (clones 10B11 and 21E10) and GAPDH were performed on iMGLs lysates from the first and the second NHD families. m) Media from NR CTRL2, HET1, HET2 and NHD3 iMGLs were collected at different times during iMGLs differentiation and sTREM2 was measured by ELISA. *P < 0.05, **P < 0.01, ***P < 0.001, Two-way ANOVA with Tukey’s post hoc test. n) Representative dot plots of SB CTRL, NHD1 and NHD2 iMGLs stained for AnxV and PI. o) relative quantification of absolute AnxV^+^ PI^+^ cells. *P < 0.5, One-way ANOVA with Kruskal Wallis test and Dunn’s multiple comparison test. Data are pooled from three independent experiments. Data shown are mean ± SEM. Supplementary Figure 3: Common pathways shared by NHD iMGLs and human macrophages. a) Cartoon representing PBMCs-derived MΦ generation from SB CTRL, NHD1 and NHD2 donors. b) NHD versus SB CTRL volcano plot of differential gene expression among iMGLs. Right, the top significant DEGs selected from DESeq2 results. The relative expression levels are represented in red-blue color scale. c) Volcano plot of DEGs among MΦ. On the right, are the top significant DEGs selected from DESeq2 results. The relative expression levels are represented in red-blue color scale. d) Pathway analysis of MΦ with down-regulated protein coding DEGs (DEG are defined by FDR < 0.05; DEG in Supplementary Table 4, online resource). e) Heatmap of DEGs in iMGLs and MΦ from SB CTRL, NHD1 and NHD2. f) Venn diagram of DEGs (NHD versus SB CTRL) shared between iMGLs and MΦ (n=345) and g) pathway analysis of DEGs shared between iMGLs and MΦ. Supplementary Figure 4: Defective activation of NHD iMGLs in resting state paralleled by an exaggerated inflammatory response after LPS challenge. a-b) Representative histograms and relative quantification showing cell surface a) CD80 and b) CD86 MFIs in NR CTRL2, HET1, HET2 and NHD3 iMGLs. c) Representative dot plots showing the % of HLA-DR^+^ iMGLs in NR CTRL2, HET1, HET2 and NHD3 iMGLs. d) Relative quantification. *P < 0.05, ***P < 0.001, ****P < 0.0001, One-way ANOVA with Tukey’s post hoc test. Data are pooled from two independent experiments. e) Representative dot plots showing the % of MERTK^+^ iMGLs in SB CTRL, NHD1 and NHD2 iMGLs and f) relative quantification. g) Representative dot plots showing MERTK^+^ iMGLs in SB CTRL, NHD1 and NHD2 iMGLs and h) relative quantification. ****P < 0.0001, One-way ANOVA with Tukey’s post hoc test. Data are pooled from two independent experiments. i) Representative confocal images of NR CTRL, SB CTRL, NHD1 and NHD2 iMGLs stained for CD68 (white), Iba1 (red) and DAPI (blue). Scale bar, 10 μm. j) Representative confocal images of NR CTRL2, HET1, HET2 and NHD3 iMGLs stained for CD68 (white), Iba1 (red) and DAPI (blue). Scale bar, 10 μm k) Quantification of CD68 area in NR CTRL2, HET1, HET2 and NHD3 iMGLs. *P < 0.05, One-way ANOVA with Kruskal Wallis test and Dunn’s multiple comparison test. Data are pooled from two independent experiments. Data shown are mean ± SEM. l-n) FACS analysis of iMGLs from SB CTRL, NHD1 and NHD2 treated for 24 h with LPS (100 ng/ml) and compared to untreated iMGLs. Graphs showing the quantification of surface a) CD80, b) CD86 MFI and c) HLA-DR^+^ cells. ***P < 0.001, ****P < 0.0001, One-way ANOVA with Tukey’s post hoc test. Data are pooled from two independent experiments. Supplementary Figure 5: Defective HLA-DR expression in NHD is restored through mTOR-dependent and independent pathways. a) Representative fluorescent images (in black and white) of NR CTRL iMGLs stained with LysoTracker Red (top). Scale bar, 10 μm. Confocal images of NR CTRL iMGLs stained with LysoTracker Red (red) and microtubule (ViaFluor® 488, green) (bottom). Scale bar, 20 μm. b) Representative confocal images of NR CTRL2, HET1, HET2 and NHD3 iMGLs stained with LysoTracker Red (top panel) or DQ-Red-BSA (bottom panel). Scale bar, 10 μm. c) Quantification of Lysotracker and d) DQ-Red-BSA intensity. ****P < 0.0001, One-way ANOVA with Kruskal Wallis test and Dunn’s multiple comparison test. Data are pooled from two independent experiments. e) Representative dot plots showing HLA-DR^+^ iMGLs in untreated or pre-treated with Torin 1 (250nM) or with the curcumin analog C1 (1 μM) in NR CTRL. f) Representative dot plots showing HLA-DR^+^ iMGLs in untreated or pre-treated with Torin 1 (250nM) or with the curcumin analog C1 (1 μM) in NR CTRL2, HET1, HET2 and NHD3. g) Relative quantification of HLA-DR^+^ iMGLs. **P < 0.01, ****P < 0.0001, One-way ANOVA with Holm-Šídák's post hoc test. Data are pooled from two independent experiments. Data shown are mean ± SEM. h) Representative confocal images of Perilipin2 (PLIN2) (green) in Iba-1^+^ NR CTRL iMGLs (red), DAPI (blue). Scale bar 10μm. i) Representative confocal images of Perilipin2 (PLIN2) (green) in Iba-1^+^ NR CTRL2, HET1, HET2 and NHD3 iMGLs (red), DAPI (blue). Scale bar, 10 μm. j) PLIN2 intensity quantification. NR CTRL2 n= 48 cells; HET1= 56 cells, HET2 n=60 cells; NHD3 n=56 cells. *P < 0.05, **P < 0.01, ****P < 0.0001, One-way ANOVA with Kruskal Wallis test and Dunn’s multiple comparison test. Data are pooled from two independent experiments. k) Representative images of Bodipy (green), DAPI (blue) staining in iMGLs. Scale bar, 10 μm. l) Relative quantification of Bodipy intensity. NR CTRL n= 24 cells; SB CTRL= 26 cells, NHD1 n=30 cells; NHD2 n=33 cells. *P < 0.05, ***P < 0.001, ****P < 0.0001, One-way ANOVA with Kruskal Wallis test and Dunn’s multiple comparison test. Data are pooled from two independent experiments. m) Representative confocal images of SB CTRL, NHD1 and NHD2 iMGLs stained with Iba-1 (cyan) and DAPI (blue) engulfing pH-rodo human myelin (red) (24h treatment). Scale bar, 10μm. n) Representative confocal images of Perilipin2 (PLIN2) (green) in Iba-1^+^ iMGLs (red), DAPI (blue) in NR CTRL iMGLs untreated or pre-treated with Torin 1 (250nM) or with the curcumin analog C1 (1 μM). Scale bar, 10μm. Supplementary Figure 6: NHD microglia are aberrantly activated and dystrophic. a) Microglia morphometric analysis in the basal ganglia (BG) of three controls and one NHD patient (NHD1^++^ (BG)). The analysis was performed using a ramification index [RI = 4π × cell area/ (cell perimeter)2] that describes microglia cell shape. BG: CTRL n= 31 cells; NHD1+ n=17. ****P<0.0001, Unpaired T-test. b) Representative images of Iba1^+^ microglia (red), PLIN2 (grey), LAMP1 (green) and DAPI (blue) staining of BG regions of a healthy control, one NHD patient (NHD1^++^ (BG)), and one MS patient (active lesion in the medulla). Scale bar, 20μm. c) Quantification of LAMP1 or I) PLIN2 signal per Iba-1^+^ cell. LAMP1 and PLIN2 intensity: BG: CTRL n= 18 cells; NHD1+ n= 20; *P < 0.05, Mann-Whitney T-test. Supplementary file1 (PDF 51519 KB)

Supplementary file2 (XLSX 770 KB)

## Data Availability

Bulk RNA-seq gene lists of iMGLs and human macrophages with statistics and NanoString gene lists of PLOS and control brains are available in Supplementary Tables 2, 4 and 6. RNA-seq data from CTRL and NHD iMGLs and human macrophages. NanoString data is currently available upon request to Dr. Wendy H. Raskind. NanoString data will be released 6 months following the publication. Human snRNA-seq data are available on Synapse (syn21125841). iPSC-iMGLs RNA-seq datasets are available on GEO (GSE157652, GSE158469). All datasets used and/or analyzed in the current study are available from the corresponding author on reasonable request.
