# Supplementary material for: Defects in lysosomal function and lipid metabolism in human microglia harboring a TREM2 loss of function mutation
Source: Acta Neuropathol. 2023 Apr 28;145(6):749–72. doi: 10.1007/s00401-023-02568-y (PMC10175346; doi:10.1007/s00401-023-02568-y)

Supplementary Figure 1

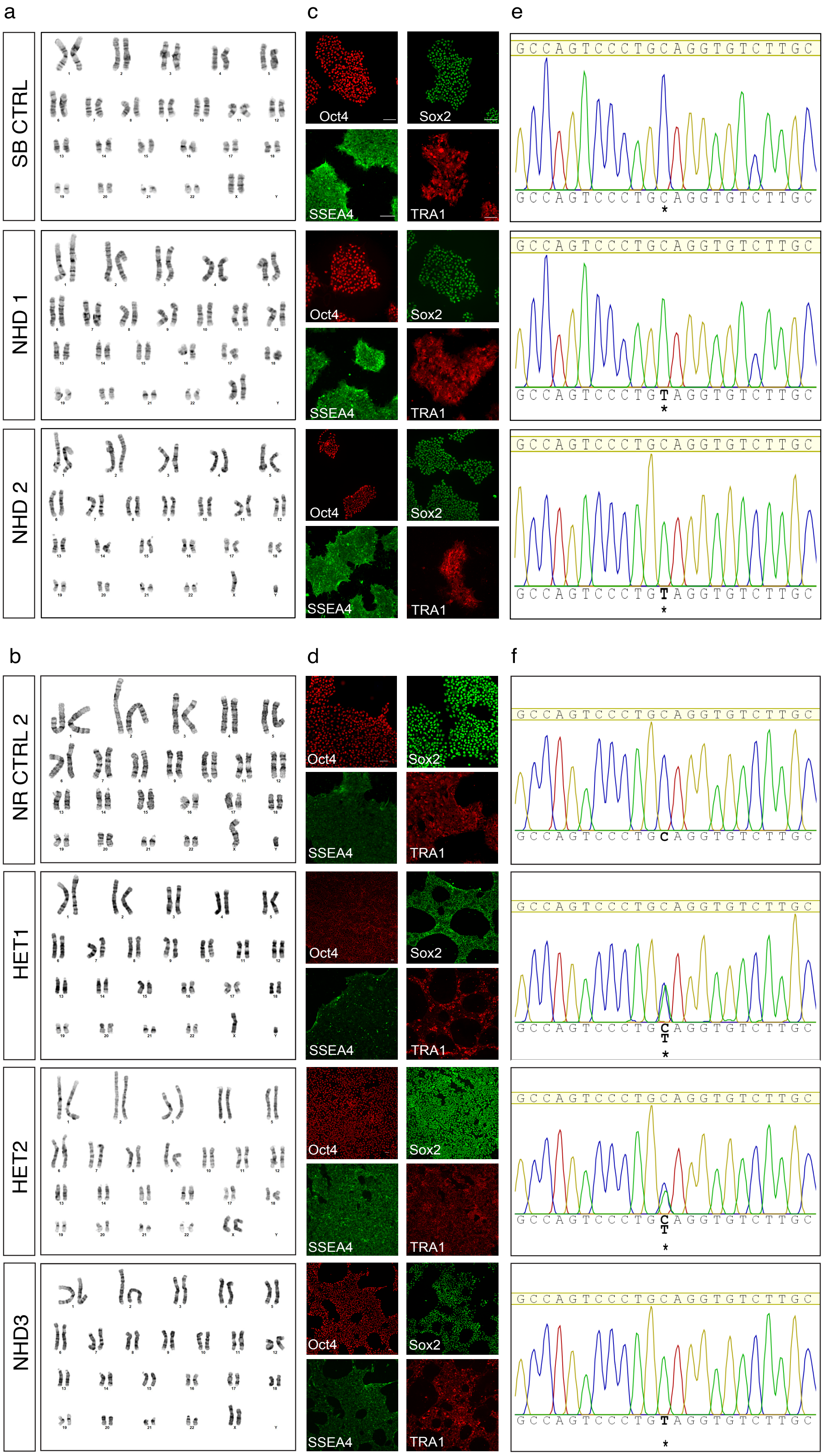

Supplementary Figure 2

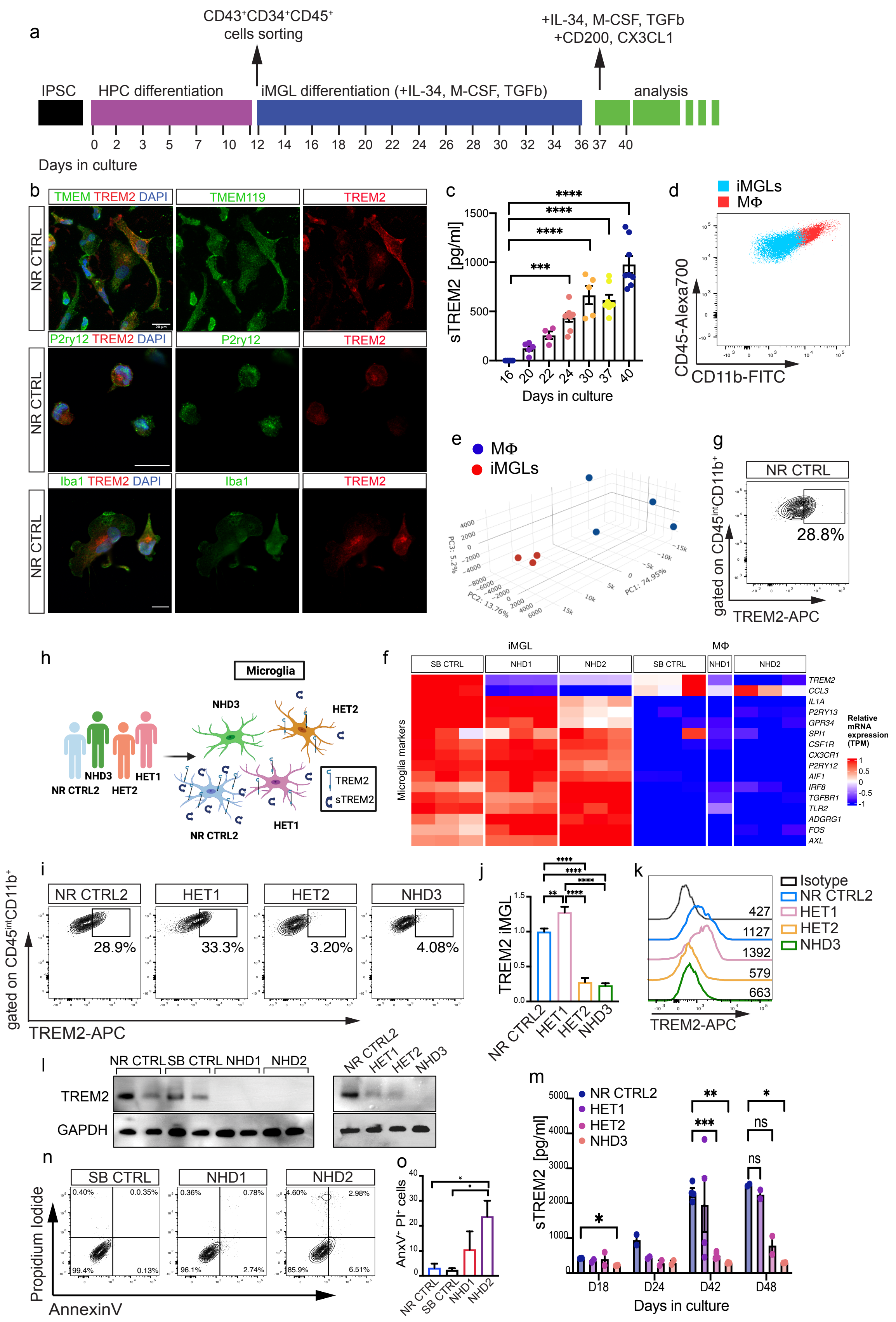

Supplementary Figure 3

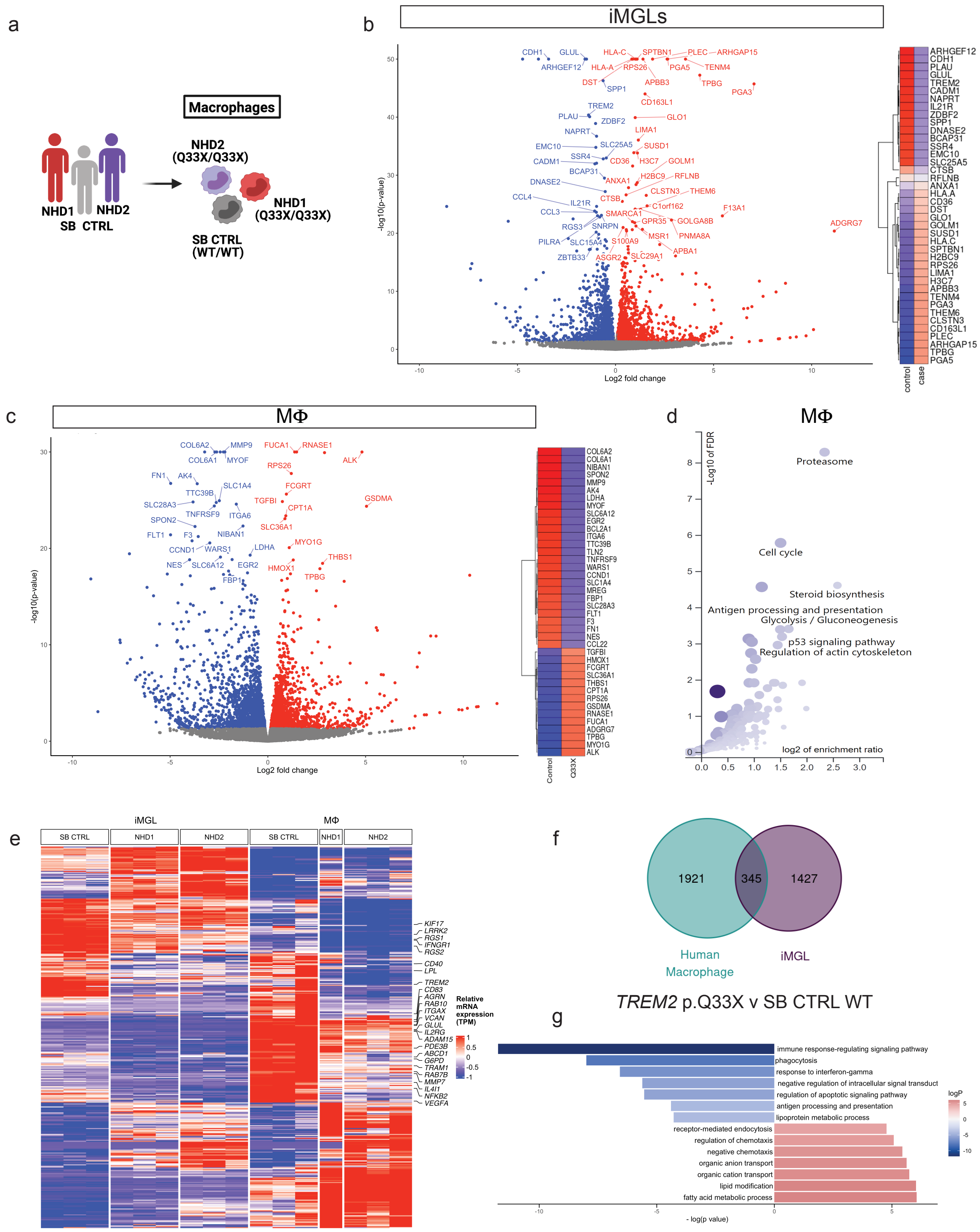

Supplementary Figure 4

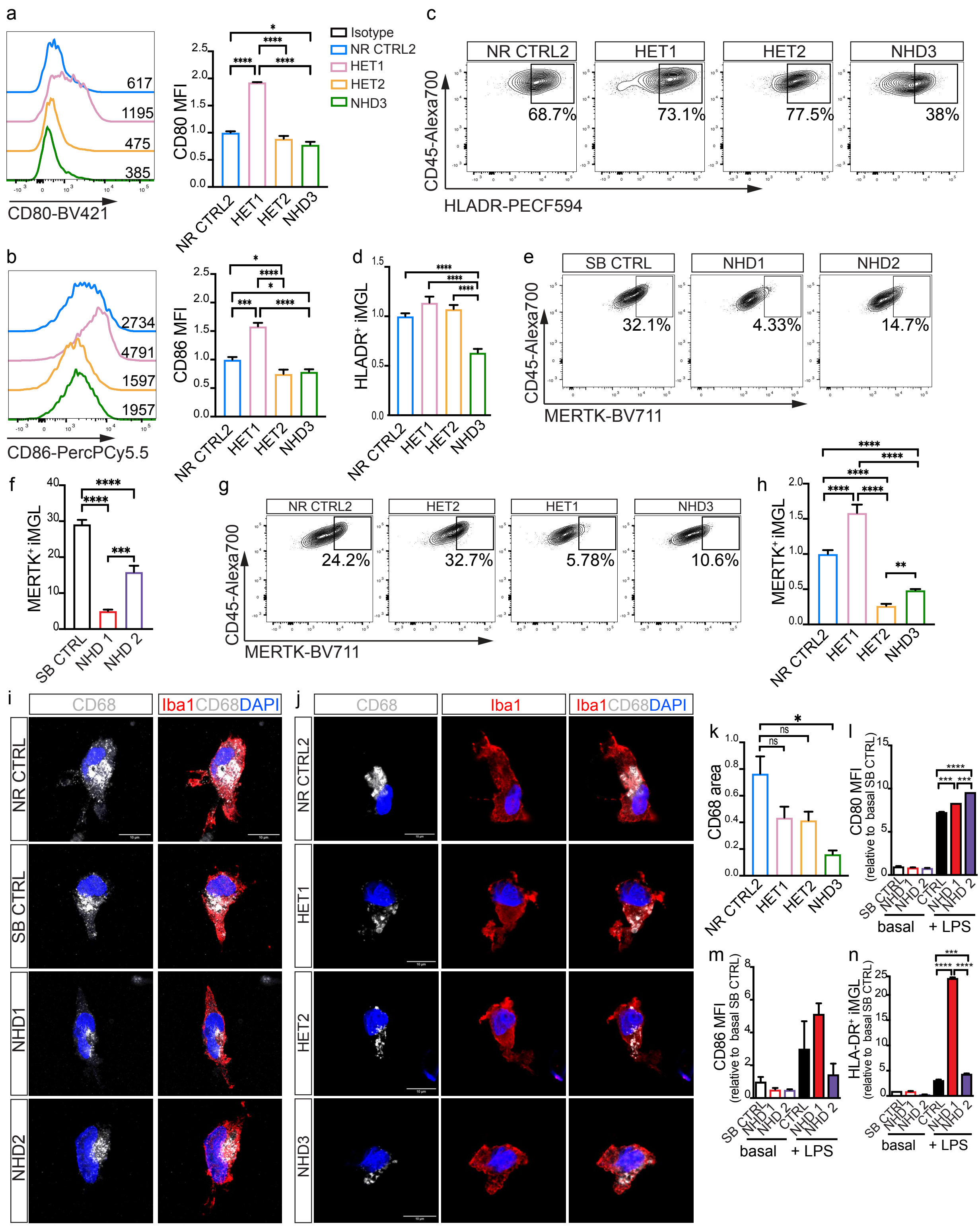

Supplementary Figure 5

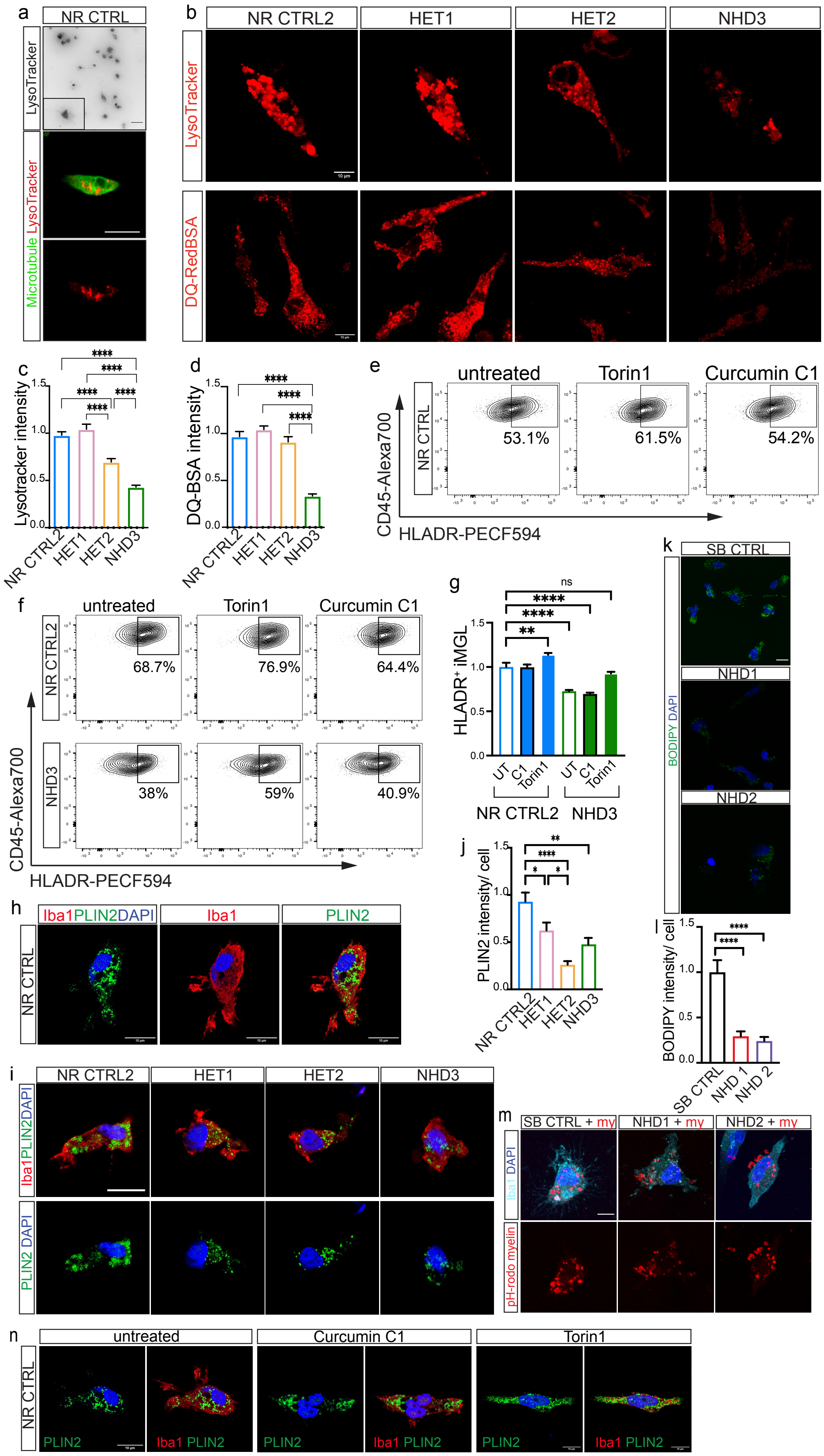

Supplementary Figure 6

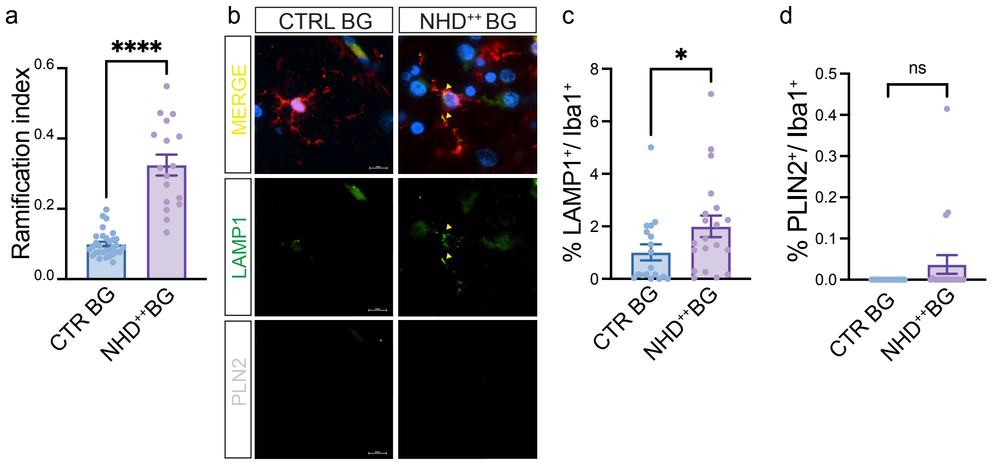

Supplement: Supplementary file 1 — Supplementary Figure 1: Generation and characterization of iPSCs from NHD patients and non-carrier family member. a-b) Karyotyping of a) iPSCs from SB CTRL and two affected siblings (NHD1 and NHD2) and b) NR CTRL2, two heterozygous parents (HET1 and HET2) and one affected child (NHD3). c-d) iPSCs were stained for octamer-binding transcription factor (OCT4), SRY-Box Transcription Factor 2 (SOX2), Stage-specific embryonic antigen-4 (SSEA4) or Transcription-associated protein 1-60 (Tra-1-60) as indicated for c) SB CTRL, NHD1 and NHD2, Scale bar, 20 μm. d) NR CTRL2, HET1, HET2 and NHD3. Scale bar, 50 μm. e-f) Sanger sequencing of TREM2 p.Q33X mutation. Sequence chromatogram showing the C to T point mutation at position 97 (C97T) in exon 2 of the TREM2 gene. *, variant site. Characterization of unrelated control iPSCs (NR CTRL) line was reported in [45]. Supplementary Figure 2: Validation and characterization of fully differentiated iMGLs. a) Schematic of iMGLs differentiation protocol (see Methods for additional information). After 40 days in culture (starting from iPSCs) iMGLs are fully mature and used for analyses. b) Confocal images of iMGLs from healthy donors (NR CTRL) stained for TMEM119, P2RY12, Iba-11 and TREM2. Scale bar, 20 μm. c) sTREM2 detected by ELISA in the media of CTRL iMGLs during the differentiation process. ***P < 0.001, ****P < 0.0001, One-way ANOVA with Dunnett’s post hoc test. Data are pooled from two independent experiments. d) Representative dot plot showing CD11b and CD45 cell surface expression in fully mature iMGLs (blue) versus macrophages (MΦ; red) from control donors. e-f) RNAseq was performed on iMGLs (N=1 SB CTRL, N=2 NHD lines; n=3 replicates/line) and MΦ (N=1 SB CTRL, N=2 NHD lines; n=3 replicates/line) (See Supplementary Table 1 for donor details, online resource). e) 3D principal component analysis (PCA) of iMGLs (red) and MΦ (blue) using the 500 genes with the highest variance (TPM normalized counts). f) Heatmap showing homeostatic [file 401_2023_2568_MOESM1_ESM.pdf]
